# Theory of Single-Impact Atomic Force Spectroscopy in liquids with material contrast

**DOI:** 10.1038/s41598-018-25828-4

**Published:** 2018-05-14

**Authors:** Enrique A. López-Guerra, Francesco Banfi, Santiago D. Solares, Gabriele Ferrini

**Affiliations:** 1 Department of Mechanical and Aerospace Engineering, The George Washington University, Washington, DC 20052 USA; 2 Interdisciplinary Laboratories for Advanced Materials Physics, Università Cattolica del Sacro Cuore, I-25121 Brescia, Italy; 3 Dipartimento di Matematica e Fisica, Università Cattolica del Sacro Cuore, I-25121 Brescia, Italy

## Abstract

Scanning probe microscopy has enabled nanoscale mapping of mechanical properties in important technological materials, such as tissues, biomaterials, polymers, nanointerfaces of composite materials, to name only a few. To improve and widen the measurement of nanoscale mechanical properties, a number of methods have been proposed to overcome the widely used force-displacement mode, that is inherently slow and limited to a quasi-static regime, mainly using multiple sinusoidal excitations of the sample base or of the cantilever. Here, a different approach is put forward. It exploits the unique capabilities of the wavelet transform analysis to harness the information encoded in a short duration spectroscopy experiment. It is based on an impulsive excitation of the cantilever and a single impact of the tip with the sample. It performs well in highly damped environments, which are often seen as problematic in other standard dynamic methods. Our results are very promising in terms of viscoelastic property discrimination. Their potential is oriented (but not limited) to samples that demand imaging in liquid native environments and also to highly vulnerable samples whose compositional mapping cannot be obtained through standard tapping imaging techniques.

## Introduction

In the last years the importance of characterizing the rheology of materials and going beyond the standard linear elastic approximation has become increasingly important. The understanding of materials whose properties are history dependent (viscoelastic materials) is becoming essential in a wide spectrum of applications. To name only a few, nanoprobing of living cells for early disease detection^1–3^, novel technological progress in energy applications such as fuel-cells^4,5^ and organic solar-cells^6^. In addition, many of these investigations, especially those on biological samples, are normally conducted in a liquid environment^7^. It is thus important to develop methods to advance the study of viscoelastic materials when the cantilever and the sample are immersed in liquids.

Dynamic atomic force microscopy (AFM) investigates the material properties gaining information from the tip-sample interaction, via the excitation of the cantilever. One important aspect of this interaction relates to the tip-sample energy dissipation, especially in inhomogeneous media, since dissipation can be used as a source of material contrast. Dissipation and material contrast are usually investigated in amplitude modulation AFM, measuring the phase-shift between the excitation signal and the cantilever response via tip-sample interaction. Phase shift is well understood in amplitude modulation atomic force microscopy (AM-AFM), both in air environment (high quality factors *Q* = 20–1000)^8,9^ and in liquid environments, with low quality factors, *Q* = 1–10, where the higher harmonic content of the tip motion is substantial^10–12^.

To go beyond single or even bimodal cantilever excitation it is possible to adopt the band excitation method, that allows an accurate mapping of energy dissipation at the nanoscale^13,14^. The frequency band is chosen to have the first cantilever resonance in the center of the excitation band. The response is analyzed, in its most straightforward approach, in terms of the Fourier transform of the cantilever deflection, fitting a simple harmonic oscillator model to determine the resonant frequency, amplitude, and Q-factor^15^.

The goal of the present theoretical analysis is to show that it is possible to go beyond Fourier analysis^16–18^ and discriminate the viscoelastic properties of heterogeneous samples using the cantilever response to a single impact of the tip^19^ on the sample’s surface in a liquid environment. Using a model that takes into account the lowest three flexural cantilever’s modes as well as the viscoelastic properties of the sample’s surface, we calculate the tip trajectory initiated by an impulsive excitation of the cantilever. The tip impacts on the surface and, due to the nonlinearity of the interaction, multiple harmonics and higher flexural modes are excited. Using the tip trajectory and the cantilever elastic stiffness as inputs, it is possible to retrieve the instantaneous values of the cantilever observables, including frequency, amplitude and phase, by an analysis based on wavelet transforms (WT). The instantaneous values of cantilever observables are uniquely associated to the viscoelastic constants of the sample material. Moreover, the instantaneous cantilever phase, being related to the surface viscoelastic properties, can be used as a source of material contrast in inhomogeneous samples. Note that there is only *one* excitation pulse for each numerical experiment (no continuous cantilever excitation), the interaction time is less than one period of the fundamental flexural mode, the excited modes are heavily damped because of the very low *Q*-factor (liquid environment). These conditions are far from optimal for the operation of an atomic force microscope in standard mode. Nevertheless, the wavelet analysis can be exploited to have an extremely rapid scanning, with a minimal dwell time, in a difficult environment as the liquid one. Acquiring material information in highly damped environments is of great interest in applications that demand imaging at liquid interfaces (e.g. studies of electrode/electrolyte interfaces in lithium-ion batteries^20,21^, imaging of surface nanobubbles^22^). This technique is also promising in providing compositional mapping of ultrasoft matter whose structure is prone to collapse after more than one impact, which would rule out standard tapping techniques.

The present method compares favorably with the available methods to measure viscoelastic constants of materials in terms of speed, frequency range and implementation. The sample is characterized with a single impulse and no averaging is needed. The viscoelastic properties are measured for short time excitations, corresponding to probing the viscoelastic properties at high frequencies, differently from the already available methods that are quasi-static or limited to few hundred kHz. Recently high-frequency microrheology evidenced important dynamics in live cells cytoskeleton^23^.

## Results

The single-impact spectroscopy method described here is performed over history-dependent (viscoelastic) materials. These samples are characterized by involved material response functions that describe their mechanical behavior during deformation, which precludes their straightforward characterization.

Here, a powerful mathematical technique (the Wavelet Transform) is exploited to gain insight regarding the discrimination of viscoelastic properties when an AFM probe impacts a viscoelastic sample in an ultrafast experiment lasting approximately one period of the fundamental cantilever oscillation. To assess the capabilities of the technique in material discrimination, numerical simulations of a cantilever impacting a wide range of viscoelastic materials are performed.

**Figure 1:**
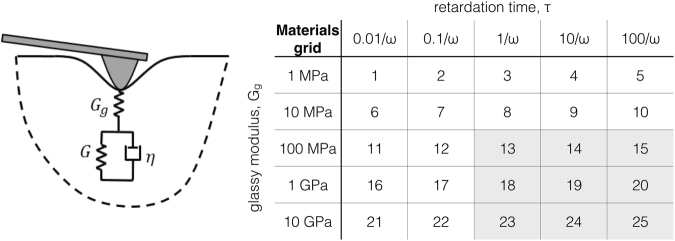
Schematic of a parabolic AFM tip penetrating a linear viscoelastic sample modeled with the standard linear solid (SLS). The geometry of the tip introduces non-linearities in the relationship between force and indentation which are very typical in AFM operation. These non-linearities are evidenced in Eq. (7) described by Lee and Radok formulation^32^ which is only appropriate for the loading portion (i.e., monotonic increase of tip-sample contact radius). In this study we have aligned to the more general Ting’s theory^24^ which has no restriction in the indentation history (more information in the Methods section). The table next to the model shows the material grid used in the simulations, where 5 values of glassy moduli (*G*_*g*_) where chosen along with 5 different retardation times (*τ*). Each value of retardation time is given in terms of the 1st eigenmode resonance frequency (*ω*). Each value of glassy modulus was combined with each of the values of retardation times, to make a total of 25 material configurations.

The viscoelastic surface is modeled with the standard linear solid (SLS), see the picture in Fig. 1. A parabolic indenter penetrating this viscoelastic solid gives rise to non-linear tip-sample force interactions, typical of AFM operation. The solution of this boundary value problem is approached numerically in the framework of Ting’s classical theory^24^ (further information in the Methods section). The material grid under study ranges a wide selection of glassy moduli, from 1 MPa (typical of soft biological samples^25^) to 10 GPa (on the scale of amorphous polymers^26^), see table in Fig. 1 (for details about the numerical simulations and modeling of the viscoelastic sample see Methods Section).

### Single-impact spectroscopy in liquids over a viscoelastic material

**Figure 2:**
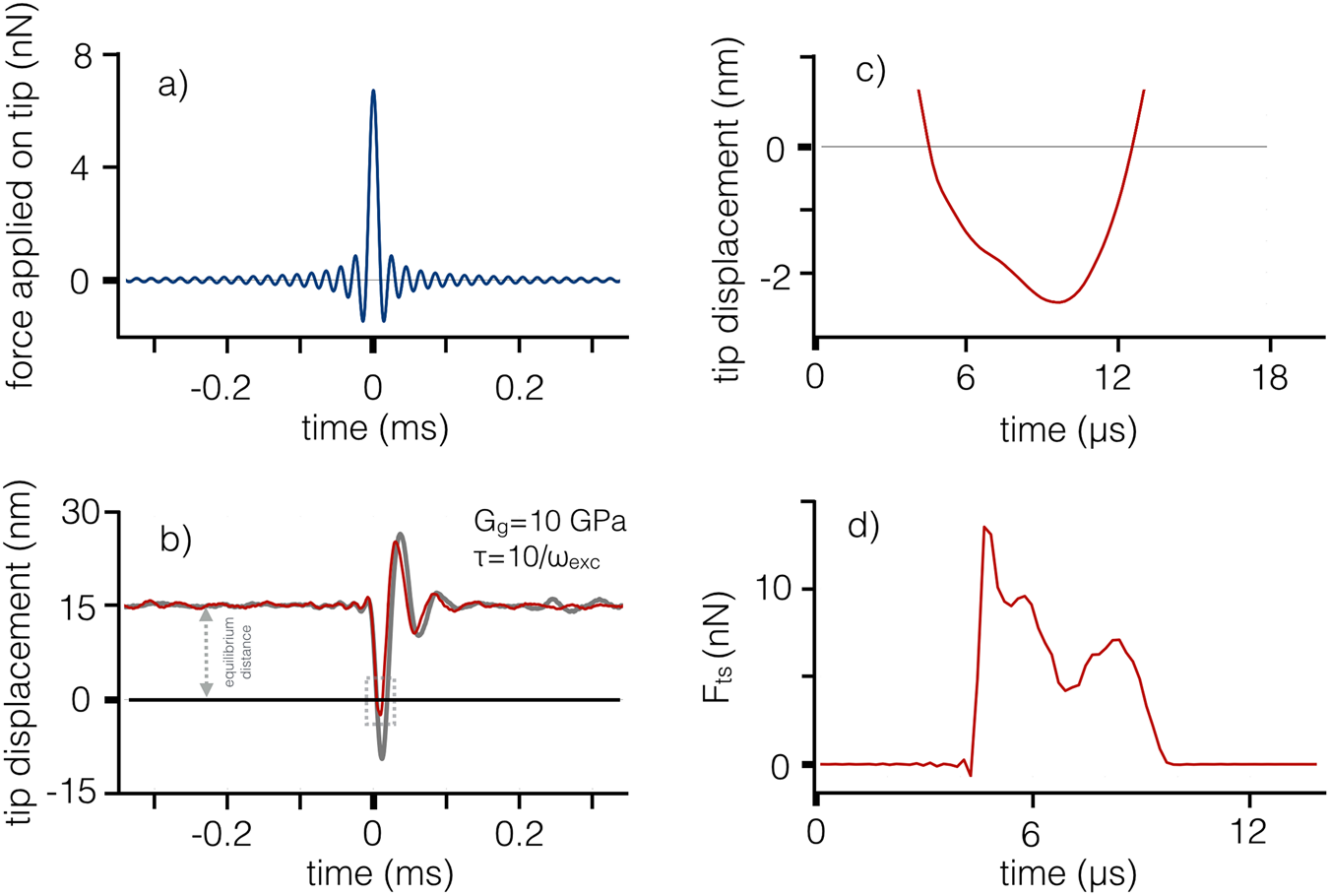
Impulsive excitation and calculated response for the tip interaction with a surface with material parameters *G*_*g*_ = 10 GPa, *τ* = 10/*ω*, where *f*_*res*_ = *ω*_*exc*_/2*π* = 20 kHz is the resonance frequency of the cantilever’s first flexural mode. (**a**) impulsive force applied to the tip in the form of a sinc function, with parameters reported in the text and the temporal zero set in coincidence with the sinc peak. Positive force applied on the tip points towards the surface. (**b**) the red trace is the calculated tip trajectory for a tip starting at an equilibrium distance of 15 nm, the surface being located at 0 nm. Note the indentation of the surface (negative displacements, dotted gray square) by the tip in the downward portion of the trajectory. The background gray trace is the free cantilever response to the same excitation, shown for comparison. (**c**) zoom of the previous graph around the indentation zone. (**d**) tip-sample force arising from the impact.

The tip interaction with the surface consists of a *single* impact measured on the time scale of the period of the fundamental cantilever oscillation. Before showing the results from the wavelet analysis of the simulations, we would like to show the timeline of events during a simulated measurement cycle. Figure 2 shows the simulations for a relatively soft cantilever in water excited with a sinc temporal profile (see Methods section for simulation parameters). The surface is characterized by a glassy modulus *G*_*g*_ = 10 GPa and a retardation time *τ* = 10/*ω*_*exc*_, where *ω*_*exc*_ = 2*π* × 20 kHz is the resonance angular frequency of the cantilever’s first flexural mode. The excitation force, shown in Fig. 2(a), is centered at time zero. In experiments, the choice of optimal excitation method in liquids is important and we will briefly discuss some alternatives in the Methods section. In the simulations the excitation force is applied directly on the tip. The tip displacement in response to the excitation is reported in Fig. 2(b), the equilibrium distance with respect to the surface is 15 nm. Note that the response oscillations have a total duration of about 100 *μ*s, corresponding to a couple of oscillations of the fundamental mode. This extreme oscillation damping is due to the very low *Q* factors of the flexural modes, appropriate for simulating a cantilever in liquids. The peak of the response oscillations is delayed with respect to the peak of the excitation sinc and indents the surface as evidenced by the zoomed detail in Fig. 2(c). Figure 2(d) shows the tip-sample force *F*_*ts*_. Both in the indentation dynamics and the tip-sample forces is present a clear modulation during the interaction time of approximately 5 *μ*s, roughly 1/10 of the resonance frequency of the fundamental flexural mode (50 *μ*s). Clearly the modulation is a consequence of the excitation of higher flexural modes, that have periods (6.8 *μ*s the second and 2.3 *μ*s the third) compatible with the observed modulation response. These higher flexural modes are responsible for modifying the observables of the fundamental mode (amplitude, phase, frequency) besides altering its energy balance. Importantly, the tip dynamics depend essentially on the viscoelastic characteristics of the indented surface and the wavelet transform is aimed at making a connection between observed dynamics and viscoelastic properties. Additionally, this excitation of higher modes could give rise to multiple impacts occurring at a single period of the fundamental oscillation. This feature can be observed in Fig. 2c and d, and is especially prominent for materials with high glassy modulus (where excitation of higher flexural modes is expected to be larger). The above feature should not be problematic, since the technique does not strictly demand a single impact. Moreover, the increase in contact time and average repulsive tip-sample force inherent to the multiple impact may vary the observables in a way that should be captured in the mapping of the model parameters space (more information about this in the materials mapping subsection). The above step is the key aspect in keeping the technique accurate despite the complexity in tip-sample interaction during this spectroscopy experiment. It is important to note that in the simulations we included the presence of thermal noise, that is responsible of the finer random features seen in the traces in Fig. 2. The inclusion of thermal noise is justified because this analysis method does not perform any average over experimental traces, as in standard AFM operations, and thus there is no mitigation of the thermal noise effects. There is, however, no adverse effect from the thermal noise on the trace analysis as will be apparent in the following discussion.

**Figure 3:**
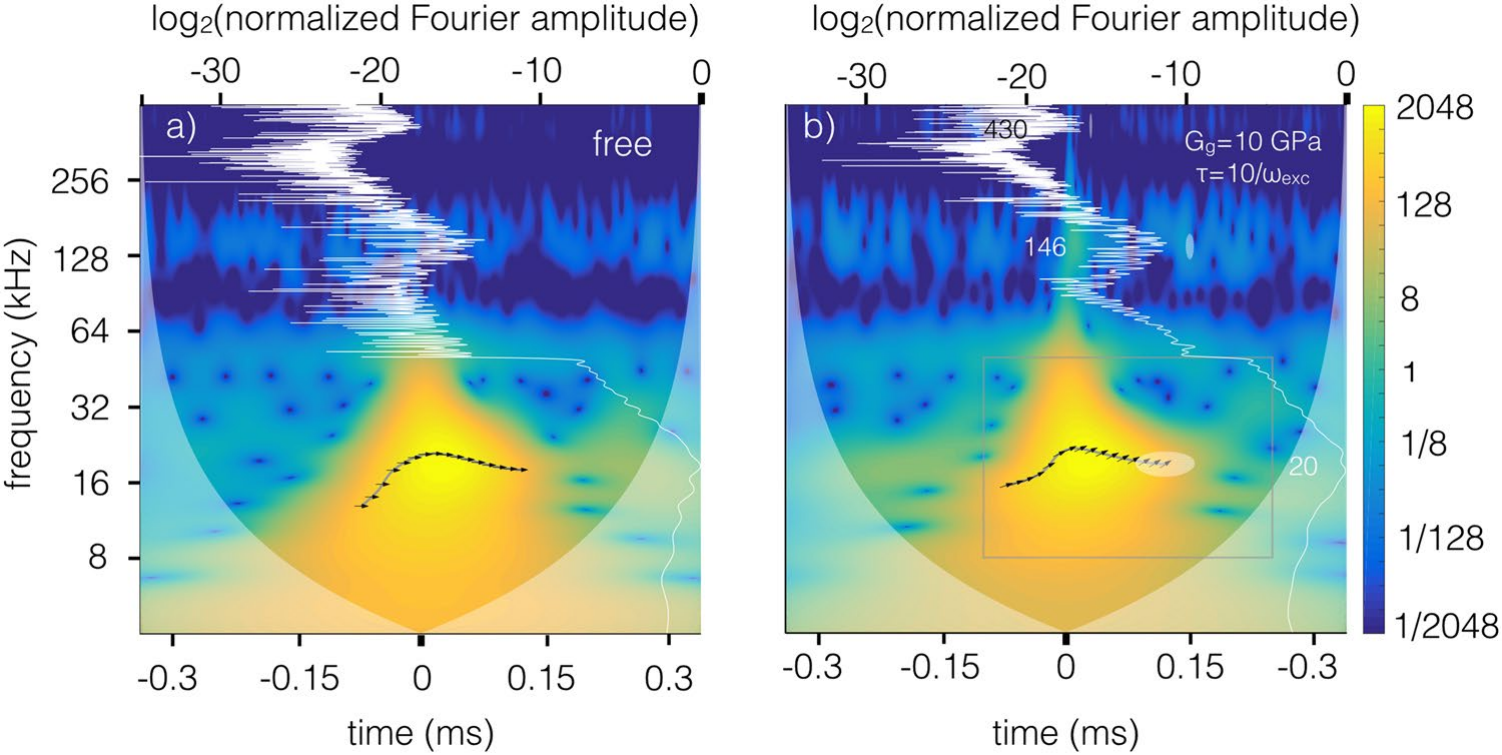
(**a**) Wavelet transform (bottom and left axis) of the tip trajectory for a free cantilever subject to the impulsive excitation force shown in Fig. 2(a). The free cantilever response is taken as a reference for the subsequent analysis. (**b**) Wavelet transform (bottom and left axis) of the tip trajectory of the interacting cantilever (shown in Fig. 2b)) after impulsive excitation, that is the signal to analyze. The rectangular box drawn by gray lines delimit the area pertaining to the first flexural mode. The three ellipses in the light white shade, drawn at the resonance frequencies of the cantilever modes, are the Heisenberg uncertainty areas, associated with the finite resolution of the Gabor wavelet used for the transform. Note that the shape of the uncertainty area changes with frequency. In both graphs, the frequency scale and the color scale are logarithmic base 2 (octaves). Edge effects become important in regions marked with a lighter shade on either side. The Fourier transform (white line, left and top axis) is superposed on the wavelet transform. The Fourier amplitude is normalized to one at its peak and the scale of the top axis shows the logarithm base 2 of the normalized amplitude. The peaks corresponding to the cantilever flexural modes are labeled with their frequency values. The gray line under the arrows is the instantaneous frequency associated with the first flexural mode during the cantilever excitation; the arrows’ rotation shows the instantaneous phase shift between signal and reference. Arrow pointing right: signal in phase with reference, phase shift = 0; left: signal in anti-phase with reference, phase shift = *π*; up: signal leading reference by *π*/2, phase shift = *π*/2; down: signal lagging reference by *π*/2, phase shift = −*π*/2.

Figure 3 shows the wavelet transform of the tip deflection traces for a free and for an interacting tip. The wavelet transform converts the one-dimensional temporal trace of the tip displacement into a two-dimensional time-frequency representation. The wavelet algorithm associates each point of the time-frequency plane with a complex coefficient, whose squared amplitude is proportional to the energy density of the signal at that specific time *and* frequency. The color bar on the right side of Fig. 3 indicates the squared magnitude of the WT coefficients. The energy density representation of a signal is called a scalogram. The scalogram provides a meaningful and intuitive representation of the temporal evolution of the spectral content of the tip’s response. Since the tip’s trajectory is influenced by the tip-sample interaction with the viscoelastic surface, its scalogram is expected to encode important material information. As a reference, the Fourier transform of the tip’s displacement is shown as a white line. The Fourier transform is not localized in time but rather averaged over the entire experimental time and therefore does not provide information on the spectral evolution of the signal in time. We show below that the wavelet transform is able to decode the wealthy information encapsulated in such a short-duration spectroscopy experiment (for details about the wavelet transform see Methods Section).

Figure 3(a) shows the wavelet transform of the free cantilever excited by an impulsive driving force shown in Fig. 2(a). The peaks corresponding to the thermally excited second and third modes are clearly visible in the Fourier spectrum. The wavelet transform shows the thermally excited higher modes as fragmented stripes running across the scalogram. Figure 3(b) shows the wavelet transform of the interacting tip’s temporal trace in Fig. 2(b). In this case the higher modes are both thermally excited, as in the previous case, and mechanically excited by the driving force through the interaction nonlinearity. The resonance frequency in kHz of the cantilever’s higher eigenmodes are written in white. The horizontal band extending across the image, in correspondence to the step in the Fourier trace, is a consequence of the finite bandwidth of the excitation. The sinc bandwidth cut around 50 kHz sets in a spectral leakage extending in time before and after time zero, a feature known as Gibbs phenomenon.

To investigate the correlation between the impulsive driver and the cantilever response it is of fundamental importance to take advantage also of the *phase* information that is encoded in the WT. Since the wavelet coefficients are complex numbers (as the Fourier transform coefficients), it is possible to define an amplitude and a phase at each point of the scalogram. In particular, it is interesting to retrieve the phase difference between the WT of two signals at each time-frequency point. This operation is technically achieved by taking the cross-correlation of the WT (XWT) of the two signals, see the Methods section for a definition of wavelet cross-correlation. If one function is seen as a reference and the other as a response to some perturbations that modify the reference signal, with the XWT it is possible to calculate the instantaneous phase shift between response and reference at all frequencies. We thus *define* “phase shift” as the phase difference between the deflection trace of the cantilever excited by an impulsive force (e.g. that shown in Fig. 2(a)) while interacting with the surface (the signal) and that of a free cantilever deflection caused by the same excitation (the reference). We emphasize that considering the phase shift as defined above, only the phase changes due to the interaction are evidenced and not those caused by the environment that are common to both signal and reference.

The knowledge of the phase shift is important because it is connected to dissipation and nonlinear interactions. As explained above, the phase shifts are calculated in principle at all frequencies and times, posing a fundamental question: which frequencies are important in the analysis of this system?

To answer this question we introduce the concept of *instantaneous frequency*. A real signal *h*(*t*), representing the tip trajectory, can be written in general terms as an amplitude modulated by a time-varying phase as *h*(*t*) = *a*(*t*) *cos* (*ϕ*(*t*)), with positive *a*(*t*). The instantaneous frequency *ω*(*t*) is defined as $\omega (t)=\frac{d\varphi (t)}{dt}$. The derivative is usually forced to be positive by a choice of the sign of *ϕ*(*t*). It is important to note that the decomposition of a generic signal into amplitude times a modulated phase is not unique. And in general, the decomposition may be of limited usefulness, especially if the signal does not have a definite carrier frequency. However, in situations where a definite carrier frequency is present, the decomposition is useful in defining a unique phase.

Using the WT it is possible to calculate the evolution of the instantaneous frequency of the signal, i.e. a trajectory in the time-frequency plane that shows how the instantaneous frequency changes in time. By taking the WT of a signal in the amplitude-phase form (*h*(*t*) = *a*(*t*) *cos* (*ϕ*(*t*))) and using an approximately analytic wavelet of the form Ψ(*t*) = *g*(*t*)*e*^*iηt*^ it can be shown that the instantaneous frequency of the signal can be retrieved from the *ridges* defined over the wavelet transform. Technically, the wavelet ridges are calculated from the normalized scalogram by finding the frequency points where the scalogram is maximum at a given time (see Methods for a definition)^27^. In other words, the ridge algorithm computes the instantaneous frequencies from the local maxima of the normalized scalogram.

The instantaneous frequency related to the first flexural mode, obtained from the wavelet ridges of the signal *h*(*t*), is shown in Fig. 3(a and b) as gray lines, describing trajectories in the time-frequency plane. Superposed on the instantaneous frequency trajectory we show the phase shift, in the meaning defined above. The phase shift is calculated along the instantaneous frequency trajectory and the result is shown by means of oriented arrows, whose rotation angle measures the local phase shift (see caption of Fig. 3). In Fig. 3(a) all arrows indicate zero phase difference since in this case the trace of a free cantilever is analyzed so that signal and reference coincide. To summarize, on the instantaneous frequency trajectory it is possible to follow the evolution of four parameters: the instantaneous frequency (left axis), the time (bottom axis), the amplitude (color), the phase shift (rotation of the arrow).

It is important at this point to comment on what it means to obtain a value for the “instantaneous” frequency, phase shift and amplitude, since wavelets are subject to a fundamental resolution limit occurring in all spectral methods (e.g. windowed Fourier transform). The WT is subject to a joint time-frequency resolution limit so that the wavelet time-bandwidth product remains constant. As a consequence, an increase in the resolution of one variable deteriorates that of the other. The light gray ellipse in Fig. 3, centered on one of the arrows displaying the instantaneous phase shift, shows the resolution of the analyzing wavelet at that point. The “instantaneous” quantities obtained from the WT are smoothed by the finite resolution of the analyzing wavelet in the time-frequency plane. Although there is no possibility to circumvents this fundamental limitation, one advantage of the Gabor wavelet is to have the smallest time-bandwidth product. It can be conveniently expressed as $\frac{{\sigma }_{T}}{T}\times \frac{{\sigma }_{f}}{f}=\frac{1}{4\pi }$, where *σ*_*T*_ and *σ*_*f*_ are the standard deviations of the time and frequency resolution limit and T is the period associated with the frequency *f* = 1/*T*. The Gabor wavelet we used to calculate the WT has a temporal standard deviation of less than one period at frequency *f*, *σ*_*T*_/*T* = 0.68 and a frequency standard deviation *σ*_*f*_/*f* = 0.116. The ellipse corresponding to the fundamental mode in Fig. 3 has the principal axes with dimension 2*σ*_*T*_ = 68 *μ*s and 2*σ*_*f*_ = 4.64 kHz at approximately the resonance frequency *f* = 20 kHz.

### Materials mapping

**Figure 4:**
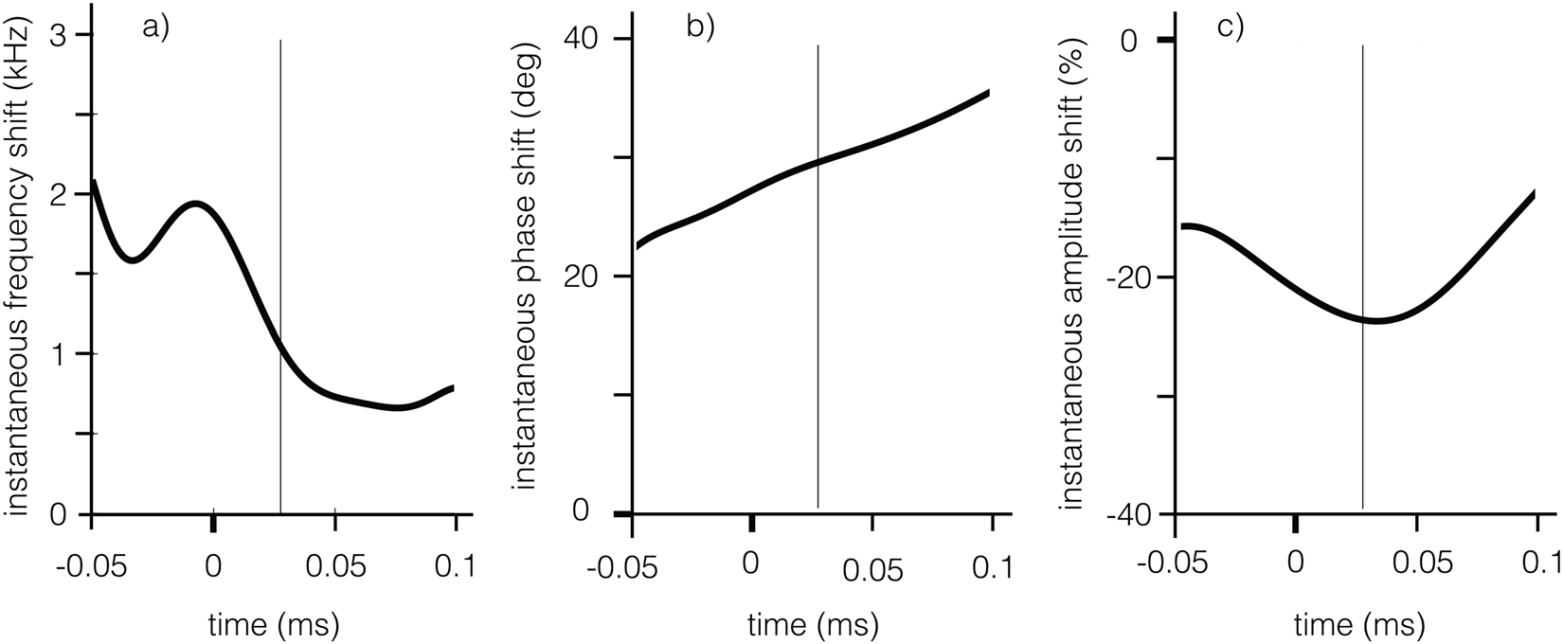
Evolution of the instantaneous parameters shifts, defined as the difference between the value for the interacting cantilever and those of the free cantilever, during tip-sample impact. Materials parameters and wavelet analysis are the same as in Fig. 3. The parameters shifts used as a metric for successive analysis in Fig. 5 are calculated at a time corresponding to the free amplitude maximum (vertical black light line).

**Figure 5:**
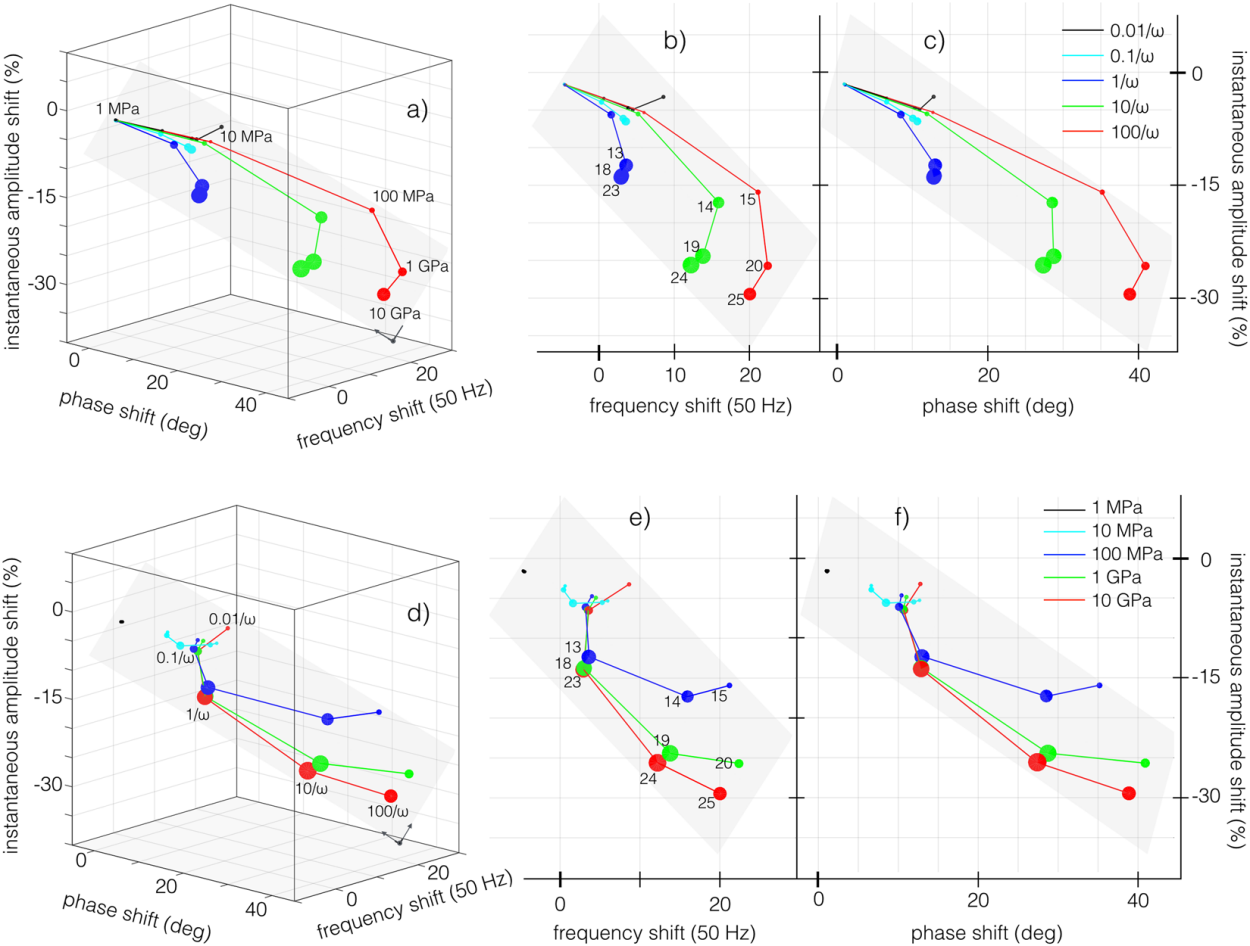
3D representations, (**a**,**d**), and projections, (**b**,**c**,**e**,**f**), of the shifts of the instantaneous parameters (frequency, phase and amplitude), with respect to the free cantilever, when the tip impacts on surfaces with different viscoelastic properties. The marker sizes are proportional to the calculated tip-sample dissipation. The points in (**a** and **d**) lay on a plane whose normal is orthogonal to the page. In (**a**–**c**) the retardation times are mapped with different colors, with the glassy modulus increasing from the origin outwards (as in the numbered sequence in panel (a)). In (**d**–**f**) the glassy moduli are mapped with different colors, with the retardation times increasing from the origin outwards (as in the numbered sequence in panel (d)). The numbers reported in (**b** and **e**) refer to the materials numbers highlighted in gray in Fig. 1.

Figure 4 shows in bi-dimensional plots the evolution of the instantaneous parameters shift with respect to an interactionless tip (frequency shift, phase shift and amplitude shift) along the instantaneous frequency trajectory of an interacting tip (see Fig. 3(b)). To extract a representative sample from the time trajectory of each parameter shift, we focus on the time instant at which the free trace shows maximum amplitude. At that instant, shown by vertical black light lines in Fig. 4, the three corresponding parameters shift are quantified. These values are used as a *metric* to characterize the investigated surface and are the ones plotted in the 3D amplitude-frequency-phase space shown in Fig. 5.

Since the phase shifts are usually given with respect to the driving force, it is worth to reconcile the observed phase shift with standard conventions. We note that the free cantilever lags the driving force in phase by *π*/2 (not shown) along the instantaneous frequency trajectory, as expected. A positive phase shift of the signal relative to the reference as observed in Fig. 4(b) means a signal lagging the driving force less than *π*/2, which is the usual condition for repulsive interaction. This metric can be used to discriminate between surfaces with different viscoelastic properties.

To implement this analysis, a 5 × 5 table is assembled by combining different values of the glassy modulus *G*_*g*_ and the retardation time *τ*, as shown in Fig. 1. The table can be thought as a discrete sampling of all the possible combinations of glassy moduli and retardation times, defining a coordinate plane. Each point on this plane is associated to a material with different viscoelastic characteristics. For each material in the table we calculate the trajectory of an interacting tip, running a simulation starting from the same initial conditions and with the same excitation waveform applied to the cantilever. The free cantilever simulation is obtained with the same initial conditions but increasing the distance from the surface to turn off the tip-sample interaction.

Figure 5 shows a representation of the quantities calculated through the WT (amplitude, phase shift and frequency shifts) for the 25 combination of parameters in the table. Interestingly, all the 25 points represented in the 3D amplitude-phase-frequency space lay almost exactly on a plane, which is skewed with respect to the coordinate axis. This fact shows that amplitude, phase and frequency are not independent and constrained to remain on a plane as the change in the viscoelastic parameters affects the dynamical conditions of the motion.

**Figure 6:**
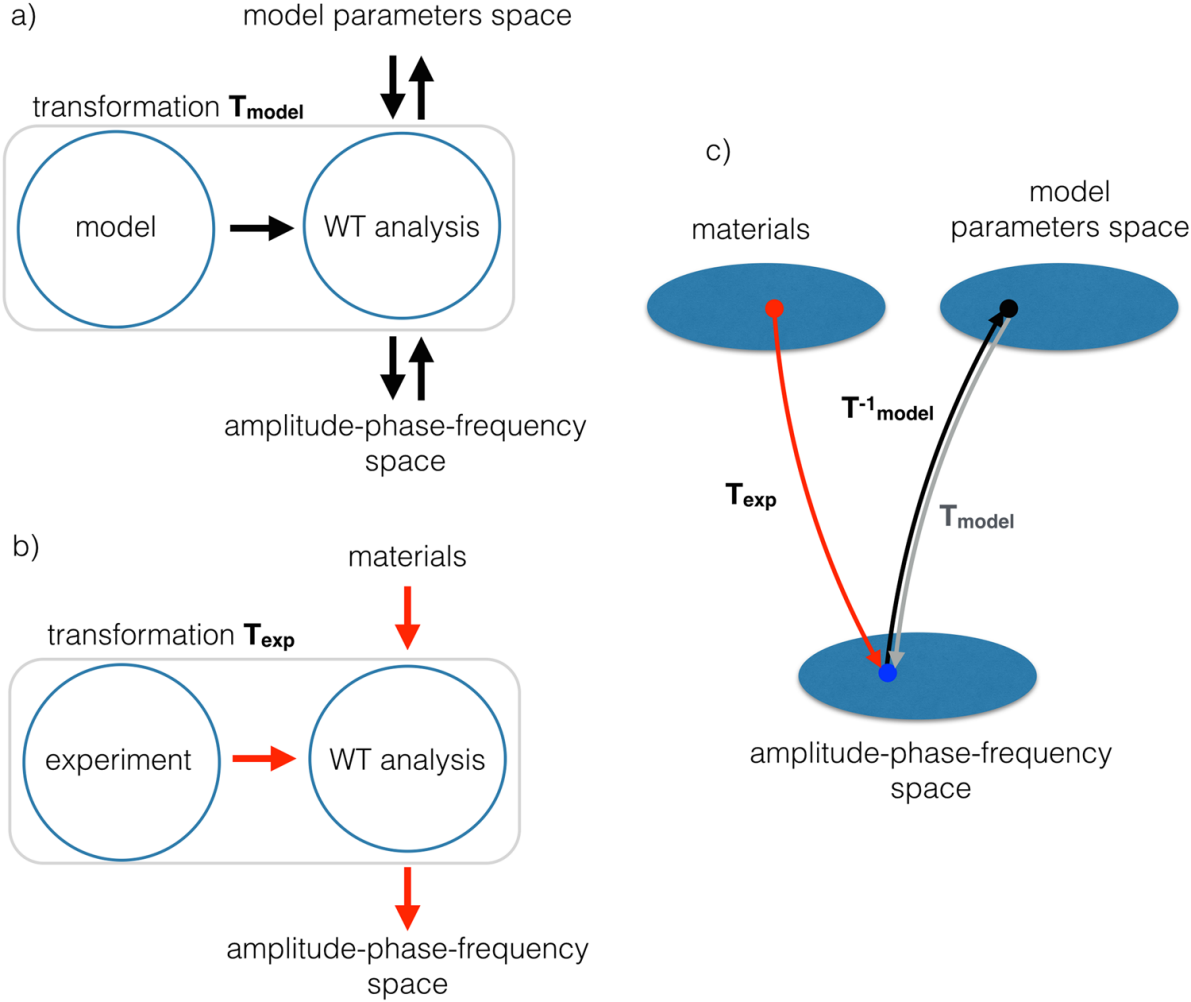
Flow charts of the main blocks of the analysis method. (**a**) representation of the mapping of the model parameters space into the amplitude-phase-frequency space. (**b**) Mapping of an experiment on a material to the amplitude-phase-frequency space. (**c**) Picture of how to associate a set of viscoelastic properties to a material using an experiment, see text for explanations.

To understand how the material properties are captured by the wavelet analysis, and how it is possible to associate a measurement to a set of viscoelastic properties, we first explain the main blocks of our analysis. Figure 6(a) shows a flow chart explaining the analysis process. The material model depends on a set of parameters that constitute the viscoelastic parameters space. A simulation of the tip impact on a surface with parameters chosen in the viscoelastic parameters space outputs a time trace of the tip deflection that constitute the input for the wavelet analysis. The wavelet analysis associate each time trace with an instantaneous amplitude, phase, frequency. This process, taken as a whole, is equivalent to a transformation **T**_**model**_ that maps the viscoelastic parameters space (domain) into the amplitude-phase-frequency space (codomain). If the transformation **T**_**model**_ is one-to-one (a bijective function), it is also possible to use the inverse function ${{\bf{T}}}_{{\bf{m}}{\bf{o}}{\bf{d}}{\bf{e}}{\bf{l}}}^{-{\bf{1}}}$ to associate each point of the codomain to one point of the domain.

The wavelet analysis can be applied to the trace generated in a real experiment. In this case the analysis process is equivalent to a transformation **T**_**exp**_ that associates an experimental trace obtained on a material with unknown viscoelastic characteristics, to a point in the amplitude-phase-frequency space, see Fig. 6(b). Assuming that the simulations reproduce the essential characteristics of the experiments, we can use the transformation ${{\bf{T}}}_{{\bf{m}}{\bf{o}}{\bf{d}}{\bf{e}}{\bf{l}}}^{-{\bf{1}}}{{\bf{T}}}_{{\bf{e}}{\bf{x}}{\bf{p}}}$ to associate the experimental trace with a point in the viscoelastic parameters space. In this way it is possible to associate a set of viscoelastic properties retrieved from the model to the investigated material, see Fig. 6(c) that illustrates this transformation.

**Figure 7:**
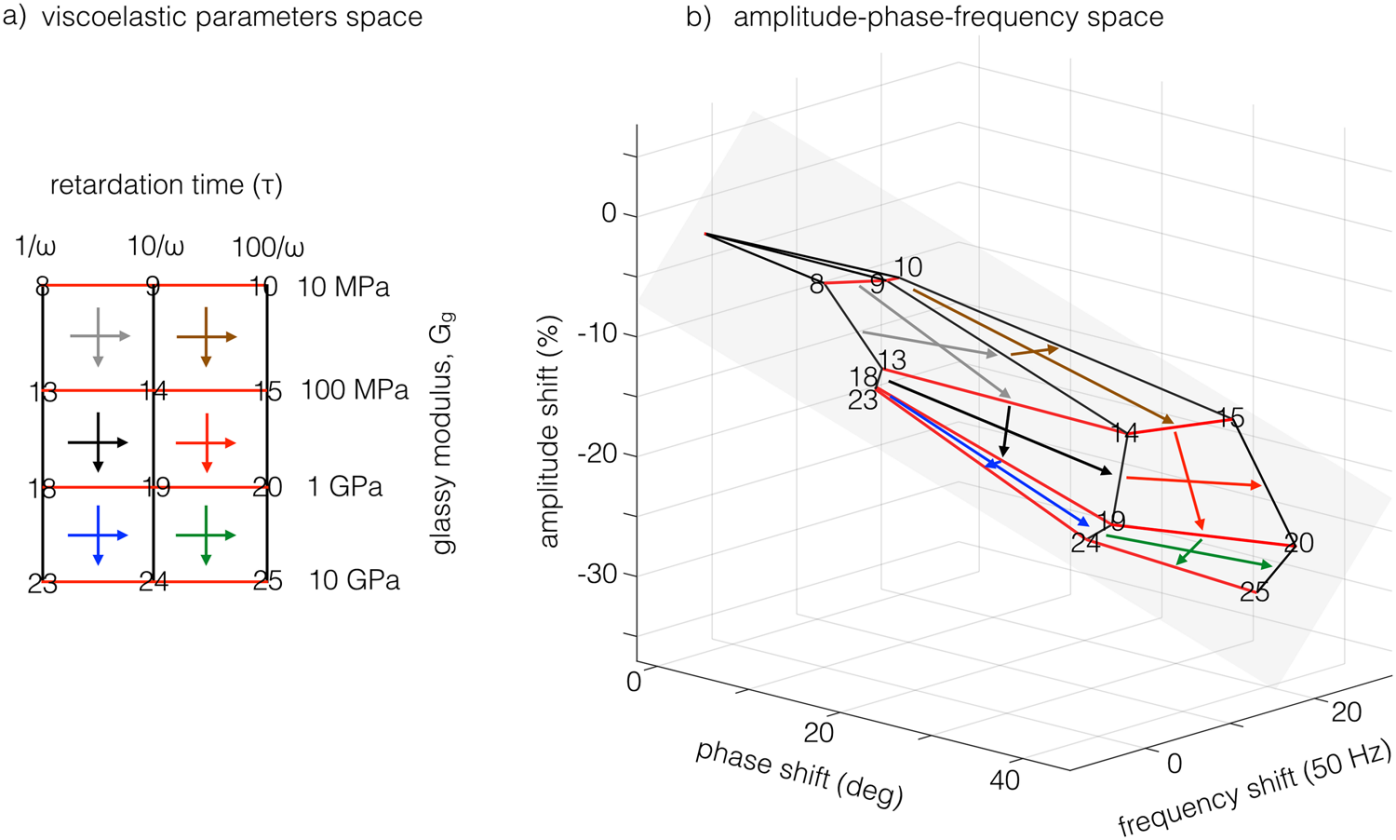
(**a**) Viscoelastic parameters space, in the form of a square grid with numbered vertices and equal logarithmic increments of the parameters. (**b**) amplitude-frequency-phase space representing the same grid as in a). Note the deformation caused by the transformation to the new coordinates and the preservation of the topology, a characteristic of transformations that behave as homeomorphisms.

As a result, we are mapping a 2D parameters space onto a plane embedded in the 3D space of the amplitude-phase-frequency space. Figure 7 shows the table in Fig. 1 as a square grid in the viscoelastic parameters space (*G*_*g*_, *τ*). Since for equal increments of the grid we have a tenfold increase of the parameters value, the representation is logarithmic. Transforming the grid points to the plane via **T**_**model**_, we obtain a deformed grid that conserve the same topology of the original, as shown in Fig. 7 using a subset of the parameters grid. The two grids, seen as deformable geometrical objects, can be superposed one onto the other by stretching and bending, a transformation equivalent to a homeomorphism. It is thus possible to define a one-to-one correspondence between the points in the materials space and the points on the embedded plane in the parameters space. The colored arrows drawn inside each quadrant point towards the direction of increasing parameters. The same lines and arrows are represented in the deformed grid in the parameters space to aid visualizing the transformation.

A desirable property of **T**_**model**_ is that nearby points in the parameters space are mapped to distant points in the amplitude-phase-frequency space, to have high sensitivity to the variation of elastic properties in heterogeneous samples. In some cases points in a region of the parameters space are mapped in a small area near a point in the amplitude-phase-frequency space, limiting the discrimination of viscoelastic properties of different materials. In such cases, it is possible to tune these properties by changing the cantilever stiffness, its dimensions and the tip-sample equilibrium distance, adapting the simulation to different ranges of viscoelastic parameters. Finally, it is clear that these method constitute the basis for a quantitative technique to characterize the viscoelastic properties of surfaces, which is rapid and can be deployed in liquid environments.

### Retrieving the time signal

The temporal signal captured through the photodiode in an AFM experiment provides the tip deflection, which has contributions from various cantilever eigenmodes, specially when the experiment is performed in low *Q* environments^10–12^. Those contributions may be decoupled by performing the inverse wavelet transform (iWT) over areas limited in frequency and time (time-frequency boxes), and centered on each eigenmode. By performing this operation, the envelope and oscillations of eigenmodes that are decoupled from the others or not strongly modified from the interaction can be retrieved with a good approximation if the signal is not corrupted from noise.

**Figure 8:**
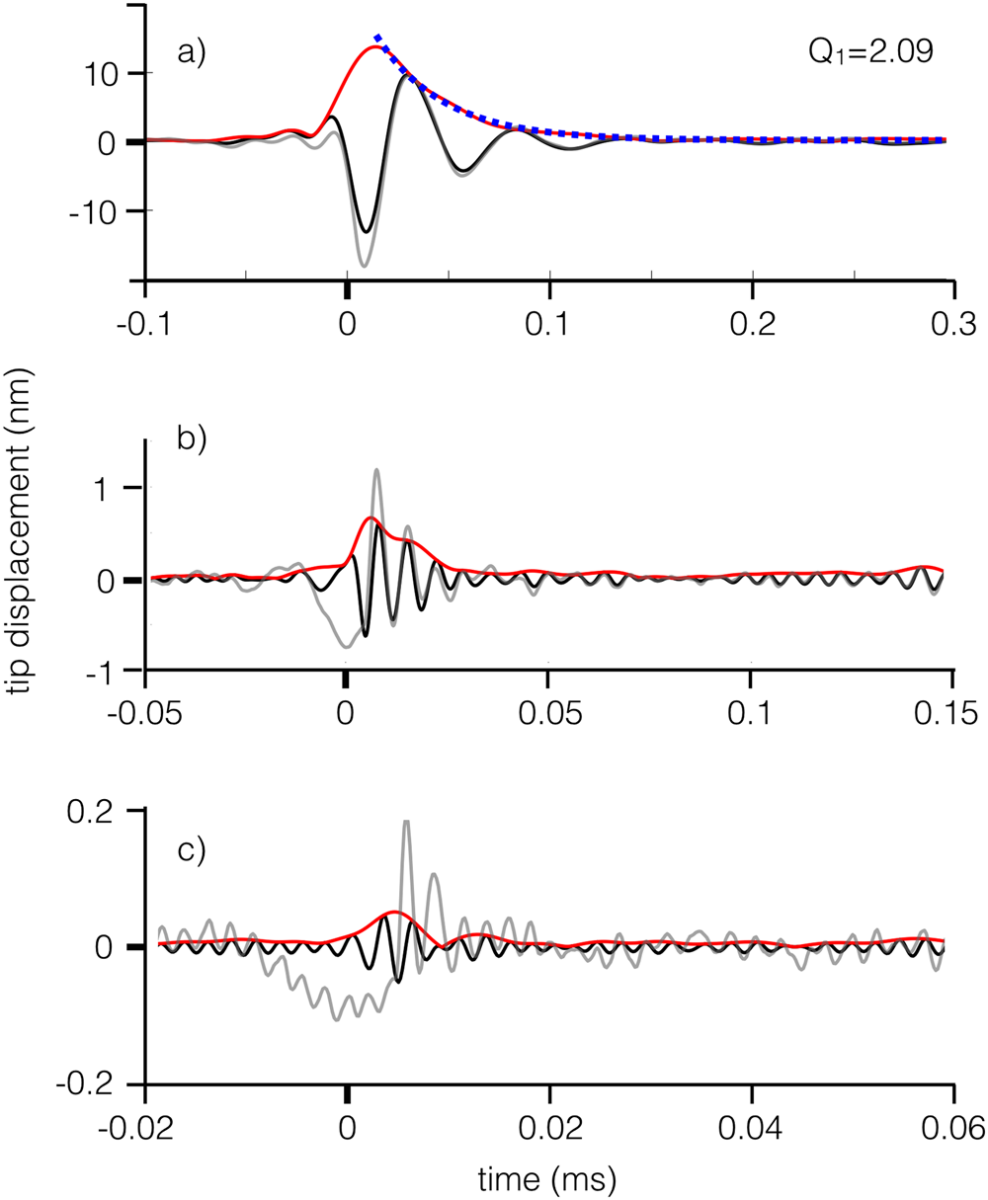
Time traces (black lines) of the first, (**a**) second, (**b**) third, (**c**) flexural eigenmodes retrieved by the inverse wavelet transform (iWT). The time-frequency intervals used to retrieve the time traces, expressed as [*t*_*min*_, *t*_*max*_, *f*_*min*_, *f*_*max*_] with time in ms and frequency in kHz, are as follows: (**a**) [−0.1, 0.3, 8, 50], (**b**) [−0.05, 0.15, 84, 256], (**c**) [−0.02, 0.06, 315, 445]. The red lines are the envelopes of the time traces. The light gray lines are the calculated time traces from the numerical simulation results. The dotted blue line in a) is an exponential fit to the decaying portion of the envelope, from which the *Q*_1_ factor is calculated. Note the scaling of times and tip displacements.

The real part of the iWT is the temporal trace of a specific eigenmode, in our case in the form of a decaying sinusoid (see Fig. 8). The absolute value of the iWT is the envelope of the eigenmode temporal trace. Figure 8 shows the temporal traces and associated envelopes of the first, second and third eigenmodes, retrieved applying the iWT to the time-frequency boxes described in the caption of Fig. 8. The time-frequency box for the fundamental mode is displayed in Fig. 3.

By fitting the envelopes with a decaying exponential function of the type exp(−*t*/*κ*), it is possible to retrieve the *Q* factor of the oscillator as *Q* = *πκf* where *f* is the carrier frequency. The *Q* factor of the fundamental mode can be retrieved via inverse wavelet transforms almost exactly because in this case the retrieved trace approximates well the calculated one. The *Q* factors from the higher modes cannot be retrieved because the signal level is too low in comparison to thermal motion. Note the rapid random oscillations caused by the thermal motion on the calculated traces in Fig. 8(b,c) The light gray lines in Fig. 8, representing the exact solutions calculated from the equation of motion for the first, second and third flexural modes. The reconstruction of the first mode is very similar to the exact solution. The calculated second and third modes have a characteristic delay and an amplitude undershoot with respect to the reconstructed oscillations. Intuitively this means that the energy transfer to the higher modes is not instantaneous and the oscillations need a couple of periods to adapt to the boundary conditions that establish the modal properties of the cantilever. These are consequences of the nonlinearity of the equation of motion, due to an unavoidable initial spectral leakage between modes. For this reason it is not possible to rely on the time traces retrieved from the iWT to calculate parameters such as the tip-sample dissipation or the tip-sample forces during the impact. In fact the iWT reconstructs the time traces using the spectrum enclosed in rectangular boxes (see caption of Fig. 8), as the one drawn in Fig. 3. Only the spectral information inside the box is used to characterize the mode, missing an important spectral component of the dynamics that is spread outside and mixed with the spectral components of higher eigenmmodes.

## Discussion

Unlike the linear elastic case, where an applied stress causes an instantaneous elastic strain (Hooke’s law), in linear viscoelastic materials stress at current time depends on the history of all strains that have been imposed at previous times. To take into account these complex relationships between stress and strain in history dependent materials, rheologists have made extensive use of spring-dashpot models^26,28,29^. The springs simulate the elastic response of the surface, while the dashpot takes into account the mechanical energy dissipation. The dashpot can be visualized as a piston–cylinder device filled with a fluid of viscosity *η*, that responds with a strain–rate proportional to stress. Among these spring-dashpot models, the standard linear solid (SLS) shown in Fig. 1 is the simplest one able to reproduce relaxation and creep behavior, which are very distinct properties observed in polymers and other viscoelastic matter. The SLS is comprised of a linear spring of shear modulus *G*_*g*_ in series with a Voigt unit. The Voigt unit is an arrangement of a spring of shear modulus *G* in parallel with a dashpot of viscosity *η*, as seen in Fig. 1. In the SLS only one retardation time is included, which is given by the ratio of the dashpot viscosity and spring modulus in the Voigt unit, *τ* = *η*/*G*. The retardation time *τ* expresses the material’s timescale, which is the time necessary for material’s rearrangements to take place upon imposition of stresses. The SLS has two effective mechanical compliances at the extremes of high and low loading rates. For loading times much shorter than the retardation time (in the limit of infinitely high loading frequencies), the dashpot behaves as a rigid body, and the total compliance is given only by the upper spring (see Fig. 1), 1/*G*_*g*_. This is the stiff-elastic regime and 1/*G*_*g*_ is the glassy compliance, which is typically orders of magnitude lower than the compliance of the spring in the Voigt unit: 1/*G*. For loading times much longer than the retardation time (in the limit of infinitely low loading frequencies) the dashpot opposes no resistance to the motion and the total compliance, in this case, is the sum of those of the two springs in series (in Fig. 1), $1/{G}_{e}=1/{G}_{g}+1/G\simeq 1/G$. This is the soft-elastic regime and 1/*G*_*e*_ is called the rubbery or equilibrium compliance (far larger than the glassy compliance 1/*G*_*g*_). This behavior of the SLS mentioned in previous lines obeys qualitatively that of amorphous polymers, which in dynamic loading tests are expected to undergo highly elastic behavior in the two extremes of frequencies. As a consequence, when the experimental timescales in applying forces to the sample are much shorter or much longer with respect to *τ*, we observe an elastic behavior (either in the stiff or soft-elastic extremes). Thus, to observe a viscoelastic behavior the experimental timescale (*τ*_*exp*_) must be of the same order of magnitude as *τ*. When this happens, material’s rearrangements are within the experimental observational window (*τ*_*exp*_) and viscoelasticity can be effectively observed. In our simulations, an AFM tip is subjected to an impulsive force excitation within a bandwidth that includes the first resonance eigenmode. Therefore, we may regard the experimental timescale as ${\tau }_{\exp } \sim 1/\omega$, being *ω* the 1st eigenmode’s resonance pulsation, which roughly governs the sample’s deformation dynamics.

When a parabolic AFM tip penetrates a linear elastic or viscoelastic sample (see Fig. 1), it introduces non-linearities in the tip-sample force *F*_*ts*_, which are typical in AFM^8^. Intuitively, the tip-sample force depends nonlinearly from indentation because, as the tip goes deeper into the surface, the contact area increases (according to the tip profile). Since each infinitesimal area element of the surface can be considered an elastic unit responding to the load force with a different compliance, an increase in contact area imply an increase in the effective compliance of the surface elastic response. For the linear elastic case the parabolic indentation solution (Hertzian) is well known and widely used in AFM literature^30,31^ (see Eq. (6)). On the other hand, the Hertzian problem for the linear viscoelastic case has been also studied since the 1960’s by several researchers (e.g., Lee and Radok^32^, Graham^33^, Hunter^34^, Ting^24^) although is not widespread in the dynamic AFM literature, being its use often reserved to static force spectroscopy^3,35–37^. Of particular usefulness (for this study) results Ting’s formulation, which is valid for the case of arbitrary indentation history. In this study, we therefore aligned to Ting’s theory, and solve numerically the viscoelastic indentation problem by following the method of dimensionality reduction^38^ (further information in the Methods section).

After having introduced key theoretical concepts, we proceed to make a semi-quantitative analysis, of the rich information retrieved from the wavelet transform method (WT), in the context of linear viscoelastic theory. Through the WT we have calculated shifts in three cantilever parameters (amplitude, phase and frequency), with respect to the parameters of a free vibrating cantilever (without tip-sample interactions). These shifts, of different simulations of the AFM cantilever interacting with 25 distinctive materials in the material grid (see Fig. 1), have been summarized in Fig. 5. The upper row (Fig. 5a–c) shows lines that connect materials with common retardation time *τ* (referred hereafter as iso-*τ* lines), while the lower row (Fig. 5d–f) shows trajectories connecting materials with the same glassy modulus *G*_*g*_ (referred hereafter as iso-*G*_*g*_ lines).

In general, shifts in the observables are small for samples with low *τ* (see in particular iso-*τ* lines: *τ* = 0.01/*ω* and *τ* = 0.1/*ω* in Fig. 5a–c) and they also display low dissipated energy *E*_*diss*_ (marker’s size is proportional to *E*_*diss*_). We relate this to a soft elastic-like behavior, governed by the rubbery modulus (1/*G*_*e*_). Qualitatively, a soft elastic-like behavior causes minor perturbations on the tip’s trajectory which translates to lower shifts in the observables.

On the other hand, we observe large shifts for samples with high *τ* with moderate *E*_*diss*_, which we relate it to a stiff elastic-like behavior. In particular see iso-*τ* line: *τ* = 100/*ω* in Fig. 5a–c. We justify this stiff elastic-like behavior because in this extreme *τ* is much longer than the experimental timescale $({\tau }_{exp} \sim \mathrm{1/}\omega )$^28^. The viscoelastic behavior is more prominent when *τ* is close to 1/*ω*, as shown by the larger markers’ size in Fig. 5a–c which indicates larger values of *E*_*diss*_ (see also Fig. S1).

We clarify that the extremes qualitatively described above as stiff elastic and soft elastic regimes are not reached within the 25 materials grid, but the trend is nonetheless respected. Moreover, moving along iso-*τ* lines we observe that *E*_*diss*_ grows monotonically, corresponding to larger *G*_*g*_ moduli which in turn cause larger tip-sample interactions favoring larger dissipations (${E}_{diss}={\int }_{0}^{T}{F}_{ts}(t)\dot{z}dt=T < {F}_{ts}\dot{z} >$, where *T* is the total time of the simulation). Phase shifts (see Fig. 5) do not follow the trends in *E*_*diss*_ as predicted by tapping mode theory in high Q environments^8,9^. We see that Δ*ϕ* increases monotonically along iso-*τ* lines (see Fig. 5c) and along iso-*G*_*g*_ lines (see Fig. 5f), which corresponds qualitatively to the behavior of <*F*_*ts*_> (Fig. S3 in Supplemental Material). This relation between Δ*ϕ* and <*F*_*ts*_> may be related to the findings of Melcher *et al*.^11^ who identified the elastic part of the tip-sample interactions as the main source for Δ*ϕ* in tapping mode with low Q environments. The justification of increasing <*F*_*ts*_> along iso-*τ* lines is consistent with the above mentioned reasoning, namely that the material moves from a pseudo-elastic soft behavior to a near-elastic stiff behavior.

Frequency shift (Δ*f*) also follows a very similar trend as <*F*_*ts*_> (as expected from standard tapping mode theory^8^), and the trends discussed in previous paragraphs are also applicable. We relate it to the negative of the virial (−<*F*_*ts*_*z*>) for the following reason. Figure 5e shows that as *τ* grows in an iso-*G*_*g*_ line, Δ*f* grows monotonically, but when following iso-*τ* lines (Fig. 5b), Δ*f* does not grow monotonically. We have seen the same qualitative behavior for the negative of the virial (see Figs S4b and S5a in the Supplemental Material), which is slightly different from the case of <*F*_*ts*_>, where the increasing monotonic behavior is consistent for all *τ* values (Fig. S5b). Further details on the viscoelastic contrast can be found in the Supplemental Material.

We highlight that the present method offers three parameters for discriminating the samples (frequency, amplitude and phase), whereas other methods either only offer one or two similar quantities (standard AM or FM) or do not provide quantitative values, or only provide averaged quantities over the entire experiment (band excitation/Fourier methods). The numerical simulations and the WT analysis show that it is possible to discriminate surfaces with different viscoelastic properties by means of experiments in which the cantilever is excited with a *single* impulse. It is important to stress that the present approach allows to discriminate materials with respect to both the glassy modulus and the retardation time, while a simple measure of dissipation could not in principle discriminate between combinations of glassy moduli and retardation times with similar dissipation. Considering that each measurement takes 0.6 ms and allowing a dead time of 0.4 ms between successive measurements, an image of 256 × 256 pixels should require approximately 1 minute and generate 1.3 GB of raw data at a sampling rate of 4 MS/s. The amount of data generated per image is sufficiently high to envision that the use of smart data analytics and data mining protocols^39^ appears particularly suited to process the data generated from imaging materials with single impact atomic force spectroscopy and will be implemented in future studies. Another interesting approach could be applying machine learning to the experimental results on different materials, along the lines of what is known as deep learning^39^, to make this kind of investigation both rapid and quantitative.

Finally, we would like to address the characteristics of the present method with respect to the already available methods that have been recently developed. To measure the viscoelastic properties of a material there are a number of possibilities based on standard force-distance curves. The viscoelastic constitutive parameters can be extracted with refined rheological models from the approach and retraction phases of standard force curves, with indentation times ranging from 25 ms to 6 sec for a 500 nm indentation on soft samples^3,36^. These measurements, highly dependent on experimental conditions, are limited to frequencies up to 40 Hz, i.e. in the quasi-static regime. By exciting the sample via a piezo-scanner it is possible to measure more rapidly the mechanical characteristic of samples at multiple frequencies up to 300 Hz, with a sensible advantage in the measurement time with respect to commercial nanoindenters^2^. In both cases the frequency is limited to the sub-kHz regime. To explore the high frequency response of soft materials, of much recent interest for the microrheological models of cells, different approaches are needed^40^. The established techniques are based on multiple frequency excitation of the cantilever, either in tapping mode for moderately stiff samples (elastic modulus <10 MPa)^41^ or in contact mode for soft samples (elastic modulus >100 kPa)^1^. In alternative, intermodulation methods have been devised, to take into account the nonlinear behavior of the interacting tip and the harmonics that perturb the cantilever resonance^42^. All these methods rely on periodic excitation of the cantilever eigenmodes, that are usually sampled for amplitude or phase in a narrow frequency interval near the resonances.

The single impact method instead propose an alternative route, by following continuously the evolution of phase, frequency and amplitude across a frequency band near resonance, taking into account the influence of the nonlinear behavior of the tip-sample interaction. The dynamical characteristics of the cantilever are not sampled at a number of distinct frequencies as in the methods described above. The instantaneous frequency is influenced by the tip-sample nonlinear interaction and local dissipation, thus constituting a set of parameters that describe intuitively the cantilever dynamics and could give quantitative information if paired with state-of-the-art viscoelastic models.

## Methods

### Wavelet transforms

The wavelet analysis is conceptually similar, though technically different, to the windowed Fourier transform. A wavelet transform (WT) is generated by the projections of a signal *h*(*t*), where *t* is time, onto a base formed by oscillating functions with zero mean and a limited support. Such functions or wavelets, ${{\rm{\Psi }}}_{s,u}(t)=\frac{1}{\sqrt{s}}{\rm{\Psi }}(\frac{t-u}{s})$, are obtained by delaying (*u*) and scaling (*s*) a mother wavelet Ψ(*t*)^27^. The projection of the signal onto the wavelets can be represented as a convolution in time or a product in Fourier space.

1 $${W}^{h}(s,u)={\int }_{-\infty }^{+\infty }h(t)\frac{1}{\sqrt{s}}{{\rm{\Psi }}}^{\ast }(\frac{t-u}{s})dt={\int }_{-\infty }^{+\infty }\hat{h}(\omega )\sqrt{s}{\hat{{\rm{\Psi }}}}^{\ast }(s\omega ){e}^{i\omega u}d\omega $$

where *ω* = 2*πf* is the angular frequency and f the frequency, $\hat{h}(\omega )$, $\hat{{\rm{\Psi }}}(\omega )$ are the Fourier transforms of *h*(*t*) and Ψ(*t*), respectively. The WT associates a complex number *W*^*h*^(*s*, *u*) to each point of the scale-delay plane (*s*, *u*). The absolute value |*W*^*h*^(*s*, *u*)| is the WT amplitude, the argument Φ^*h*^(*s*, *d*) = arg(*W*^*h*^(*s*, *u*)) is the WT phase. The equivalent Fourier period at scale s can be derived analytically for a particular wavelet function following the method in reference^43^. We use as mother wavelet the Gabor form ${\rm{\Psi }}(t)=\frac{1}{{(\pi )}^{\mathrm{1/4}}}\exp \,(-\,\frac{{t}^{2}}{2}+i{\omega }_{0}t)$, where *ω*_0_ is the carrier frequency. In this case, the scale-frequency relation in the WT is to a very good approximation equal to $f=\frac{{\omega }_{0}}{2\pi s}$.

The instantaneous frequency is measured from ridges defined over the wavelet transform. The wavelet ridges are the points corresponding to the position of the maxima of the normalized scalogram |*W*^*h*^(*u*, *s*)|^2^/*s* at each *u*. The maximum position at each *u* determines the scale as a function of *u*, *s*(*u*). The set of points (*u*, *s*(*u*)) is by definition a ridge.

A fundamental operation on the WT is the cross-correlation of two functions *h*(*t*) and *r*(*t*)^44^. Cross-correlation is obtained by multiplying *W*^*h*^(*s*, *u*) by the complex conjugate transform of *r*(*t*), *W*^*hr*^ = *W*^*h*^(*s*, *u*)*W*^*r*^(*s*, *u*)^*^ = |*W*^*h*^(*s*, *u*)||*W*^*r*^(*s*, *u*)| exp (*i*Φ^*h*^(*s*, *u*) − *i*Φ^*r*^(*s*, *u*)). From the cross correlation it is possible to retrieve the instantaneous phase difference between two functions at every point (*s*, *u*). If the function *h*(*t*) is seen as a driver (or cause) and *r*(*t*) as a response (or consequence), from the cross-correlation it is possible to calculate the instantaneous phase shift between driver and response.

The signal *h*(*t*) can be retrieved from the WT using the following inverse relation (iWT):

2 $$h(t)={Re}[{h}_{a}(t)]={Re}[\frac{2}{{C}_{{\rm{\Psi }}}}{\int }_{0}^{+\infty }{\int }_{-\infty }^{+\infty }{W}^{h}(s,u)\frac{1}{\sqrt{s}}{\rm{\Psi }}(\frac{t-u}{s})du\frac{ds}{{s}^{2}}]$$

where *h*_*a*_(*t*) is the (complex) analytic signal associated with *h*(*t*) and *C*_Ψ_ is a real constant for normalization purposes^27^. The inverse wavelet transform that uses an analytical mother wavelet Ψ(*t*) (or approximately so as the Gabor wavelet), produces a complex signal that is the analytic signal *h*_*a*_(*t*) associated with *h*(*t*). The complex analytic signal can be represented by a modulus and a phase. The modulus of the analytic signal |*h*_*a*_(*t*)| is the analytic amplitude or envelope of *h*(*t*). The time derivative of the phase is the instantaneous frequency. Both envelope and instantaneous frequency are uniquely defined through the analytic signal generated by *h*(*t*). Through the iWT it is possible to obtain an analytic signal associated with the inverse transform of a limited area of the time-frequency plane. Thus it is possible to retrieve the temporal trace and envelope of a single excited eigenmode by performing the iWT only on a delimited area pertaining to that mode, as illustrated by the rectangular box drawn with gray lines in Fig. 3b.

The approach based on wavelet transforms may be applied to mechanical nanometrology techniques other than AFM. For instance, impulsive time-resolved techniques^45,46^ are the high frequency analogue–with mechanical frequencies in the GHz to THz range–of single impact atomic-force spectroscopy and may thus benefit from the analysis methods developed here.

### Numerical Simulations

The dynamics of the cantilever tip are assumed to be mainly contained in the lower modes and therefore we included only the contribution of the first three flexural eigenmodes, using an individual equation of motion for each of them, all coupled through the tip-sample interaction forces. The equation of motion for the three flexural modes was the following:

3 $$m{\ddot{z}}_{i}(t)+\frac{m{\omega }_{0i}}{{Q}_{i}}{\dot{z}}_{i}(t)+{k}_{i}{z}_{i}(t)=f(t)+{ {\mathcal F} }_{ts}[{z}_{s}(t,u),d]+{F}_{{B}_{i}}(t)$$

where *m* is the effective mass of the cantilever, *z*_*i*_, *k*_*i*_, ${\omega }_{0i}=2\pi {f}_{{0}_{i}}$, and *Q*_*i*_ refer to the *i*_*th*_ eigenmode’s (with *i* = 1, 2, 3) displacement, force constant, resonance frequency, and quality factor, respectively. ${ {\mathcal F} }_{ts}$ represents the tip-sample forces, *z*_*s*_ the sample deformation and *d* the tip-sample distance. The cantilever parameters in liquid have been chosen as *k*_1_ = 0.25 N/m, *k*_2_ = 9.8 N/m, *k*_3_ = 77 N/m, *f*_01_ = 20 kHz, *f*_02_ = 146 kHz, *f*_03_ = 429 kHz, *Q*_1_ = 2, *Q*_2_ = 4.4, *Q*_3_ = 6.8, R = 10 nm, where R is the tip radius of curvature. These values are representative of a cantilever with a moderately high resonance frequency in liquid and similar values are found in the literature, e.g. see ref.^12^. ${F}_{{B}_{i}}(t)$ is a stochastic Brownian force that takes into account the interaction of the cantilever with the thermal bath for each mode. *f*(*t*) is the external excitation force applied on the tip.

The excitation function, *f*(*t*), applied to the tip is chosen to be the sinc function, and is defined as^47^

4 $$A\,{\rm{sinc}}(\frac{t-{t}_{0}}{a})=A\frac{\sin \,(\frac{t-{t}_{0}}{a})}{(\frac{t-{t}_{0}}{a})},$$

where *A* = 0.6 nN is the peak amplitude and a is a shape parameter that controls the width of the function centered at *t*_0_ and its bandwidth. We choose a bandwidth BW = 2.5*f*_1_ so that $a=\frac{1}{2\pi BW}$.

The notation employed to represent the tip-sample force term ${ {\mathcal F} }_{ts}$ in Eq. 1 emphasizes the nature of the viscoelastic material used in the simulation. According to it, the tip-sample force *F*_*ts*_ is a functional of the sample deformation, i.e., the force at the current time *t*, *F*_*ts*_(*t*), depends on the history of the surface deformation at all past times *u*^28^. Since the dynamics of the tip are assumed to be mainly contained in the first three modes, the tip position becomes $z(t)={\sum }_{i=1}^{3}{z}_{i}$. The total energy dissipated through the tip-sample interaction can be calculated through decomposition of the contribution of each eigenmode as^8^ ${E}_{ts}(i)={\int }_{0}^{T}{F}_{ts}(t)\frac{d{z}_{i}(t)}{dt}dt$, where *T* is the total time of the simulation. The total dissipated energy is given by ${E}_{ts}={\sum }_{i=1}^{3}{E}_{ts}(i)$.

The spectral density of the Brownian force ${F}_{{B}_{i}}(t)$ for each mode *i* is derived from the fluctuation-dissipation theorem. The Brownian force spectral density in the proximity of each mode is ${S}_{{F}_{{B}_{i}}}(f)=4{k}_{B}T{\gamma }_{i}(f)$, where *γ*_*i*_(*f*) is a mode dependent dissipation parameter, *k*_*B*_ is the Boltzmann’s constant, *T* is the absolute temperature. For a damped harmonic oscillator *γ*_*i*_(*f*) = *m*_*i*_*ω*_*i*_/*Q*_*i*_, where *m*_*i*_ is the total mode mass, taking into account the added fluid mass, *ω*_*i*_ = 2*πf*_*i*_ and *Q*_*i*_ are the radial resonance frequency and quality factor for each mode *in liquid*. Using the relation between the vacuum frequency ${\omega }_{i}^{vac}$ and the resonant frequency in liquid ${m}_{c}{({\omega }_{i}^{vac})}^{2}=m{\omega }_{i}^{2}$, it is possible to express the cantilever spring constant ${k}_{i}={M}_{e}{m}_{c}{({\omega }_{i}^{vac})}^{2}$ in terms of the mass and frequency in liquid ${k}_{i}={M}_{e}{m}_{i}{\omega }_{i}^{2}$, where *M*_*e*_ is the modal effective mass, equal to 0.25 for all the modes^48,49^. The dissipation parameter is thus expressed as ${\gamma }_{i}(f)=\frac{{k}_{i}}{{M}_{e}{Q}_{i}{\omega }_{i}}$, involving only cantilever parameters measurable in liquid for each mode.

From the spectral density of the Brownian force it is possible to obtain a stochastic time-series of the Brownian force for each mode:

5 $${F}_{{B}_{i}}(t)=\sum _{k=1}^{n/2}2\sqrt{{S}_{{F}_{{B}_{i}}}({f}_{k}){\rm{\Delta }}f}\,\cos \,(2\pi {f}_{k}t+{\theta }_{k})$$

where *n* is the total number of samples, ${\rm{\Delta }}f=\frac{1}{n{\rm{\Delta }}t}$, Δ*t* being the sampling time, *f*_*k*_ = *k*Δ*f* and *θ* is a stochastic variable distributed between 0 and *π*.

In adding the Brownian force to the equations of motion, the following approximations where made: (1) each mode is treated as a damped harmonic oscillator^50^ with parameters *f*_*i*_, *Q*_*i*_, *k*_*i*_ in liquid; (2) we do not take into account the influence of the sample surface on the cantilever motion (the so called film squeezing effects)^51,52^; (3) the shape of the eigenmodes of the cantilever is not modified by the hydrodynamic loading, so that *M*_*e*_ is mode independent^53^; (4) the Brownian force spectral density is not white, i.e. constant over the modes, but dependent on the measurable parameters of each mode, as expressed above. This approximate the frequency dependent Brownian force spectral density as constant around each mode.

As consequence of these approximations, the motion of the cantilever is treated as in an infinite viscous fluid, where only the inertial (added mass) effects are taken into account. This is a good approximation provided the beam distance from the surface is equal or greater than the cantilever’s width^52^. The approximation on the Brownian force density is adequate for our purposes and more sophisticated approaches could be devised if needed.

### Modeling of indentation

Indentation of elastic surfaces by axisymmetric indenters has been exhaustively studied and the solutions are widely known. The specific case of a rigid parabolic indenter penetrating an elastic sample is given by^30,31^:

6 $${h}^{3/2}=\frac{3(1-\nu )}{8\sqrt{R}}JF$$

where *F*, *R*, and *h* are the force, radius of curvature of the tip apex and surface displacement, respectively; *J* and *ν* are sample’s elastic constants: shear compliance and Poisson’s ratio, respectively.

On the other hand, indentation of viscoelastic surfaces by rigid indenters has been also thoroughly studied^24,32–34^. Initially, Lee and Radok^32^ demonstrated that the Hertzian problem for linear viscoelastic materials was appropriately deduced from its elastic counterpart:

7 $$h{(t)}^{3/2}=\frac{3}{16\sqrt{R}}{\int }_{0}^{t}J(t-\xi )\frac{dF(\xi )}{d\xi }d\xi $$

where *h*(*t*) is the surface displacement, *R* is the tip radius, *J*(*t*) the shear creep compliance (shear strain response to a unit step shear stress^54^), and *F*(*t*) is the force applied to the viscoelastic surface. This convolution integral results from the direct application of the elastic-viscoelastic correspondence principle^55^ to the linear elastic solution^30,31^. However, they pointed out that it was only applicable for cases of monotonic increase contact radius.

A further extension which does not have the above restriction was derived by Ting^24^ for arbitrary loading histories, a method that has been contemporarily revisited and simplified by Greenwood^56^. The above methods in general lead to relatively complicated convolution integrals, whose difficulty in their evaluation is related to the level of detail with which the material functions (e.g., shear compliance) are described, and on the deformation history of the material (i.e. number of maxima and minima in the contact radius history). Those material functions are complex because the mechanical response of viscoelastic materials is typified by inclusion of multiple characteristic times in which rearrangements occur upon deformation. These rearrangements are observed as relaxation or retardation (creep) responses upon strain or stress excitations. The above distinct features have encouraged rheologists to use sophisticated arrangements of spring dashpot models, continuous spectral representations, ladder models, etc^28^. to take into account the intricate physics in the mechanical deformation of these materials. The above intricacies seem to have hindered the applicability of those early solutions in other contemporary research areas related to dynamic nanomechanical probing (e.g., dynamic AFM).

In AFM often the physics has primarily focused on the cantilever dynamics with little attention given to inclusion of sophisticated models able to mimic the deformation of viscoelastic materials in a realistic way. A common strategy has been to use the Voigt model (an arrangement of a spring and a dashpot in parallel), a very simple model only capable of displaying retardation (creep) but not relaxation phenomena. The Voigt model is regarded in polymer rheology as simply a building block in more sophisticated models (therefore it is often called Voigt unit). Lately some efforts have been spent to incorporate more thorough viscoelastic descriptions in modeling dynamic AFM^57,58^ with inclusion of multiple relaxation times and their relation to observables through analytical expressions^36,59^.

The challenge in solving explicitly Ting’s convolution integrals^24^ (which requires *a priori* knowledge of the force, displacement, or contact radius history) can be circumvented by implementing continuum simulations using approaches such as the finite element model (FEM) to solve the three-dimensional problem of a tip penetrating a viscoelastic surface^60^. The above, although precise, is computationally expensive. An interesting alternative arose contemporaneously with the method of dimensionality reduction (MDR)^38^. In this method, Popov and Heß^38^ substitute a three-dimensional continuum by a uniquely defined one-dimensional linearly elastic or viscoelastic foundation (Wrinkler foundation). This method is relatively inexpensive computationally and easy to implement by following a simple users’ handbook written by the authors^61^. Although seemingly simple, this method has proven to generate exact solutions for the general viscoelastic indentation problem^62,63^. For the above reasons, we have used this method for our simulations in this study.

### Implementation of the single-impact approach in a force microscope

**Figure 9:**
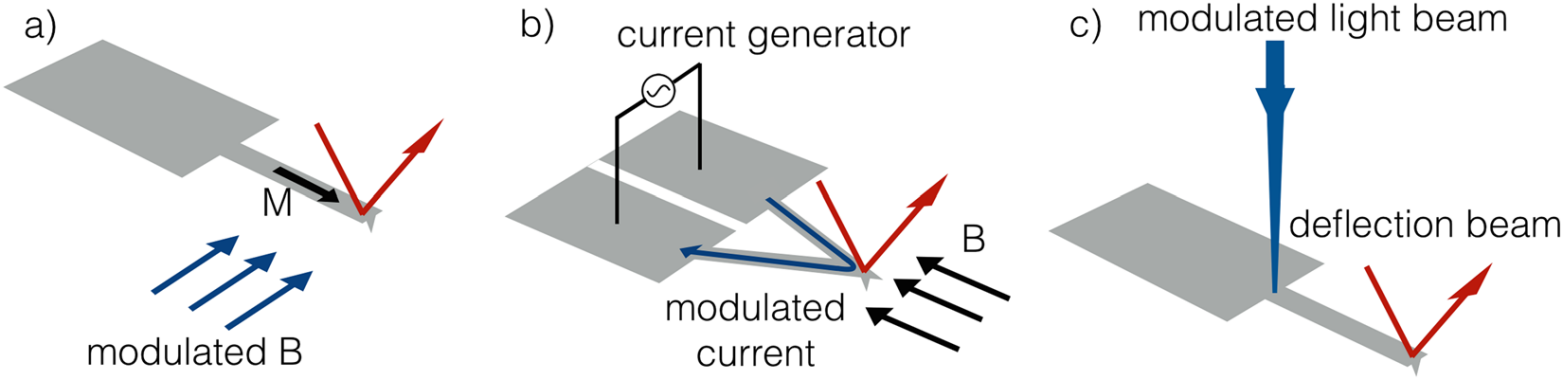
Excitation methods for the cantilever.

In this section we discuss the implementation of the single-impact spectroscopy method in an AFM microscope, where the cantilever is excited by applying an impulsive force on the tip. There are mainly two types of cantilever excitations that are particularly adapted to fluids: magnetic excitations and photothermal excitation. The standard piezoacoustic excitation at the base of the cantilever is not the method of choice in liquids, due to the parasitic resonances it introduces in the excitation spectrum of the cantilever. Parasitic resonances are mainly due to the energy delivered to the fluid in form of waves by the excitation of the cantilever base, that are subsequently scattered onto the cantilever and alter its motion^64^. Magnetic excitation solves the problems by applying the driving force right on the cantilever’s tip by a modulated magnetic field or a modulated current via Lorentz forces. There are basically two widely used magnetic excitation schemes. In Fig. 9(a) the cantilever beam is covered with a thin film of magnetic material magnetized along the beam axis. In alternative only the tip can be magnetized. A modulated magnetic field *B*, e.g. from a nearby solenoid, with components orthogonal to the magnetization *M* provides a bending moment $\overrightarrow{\tau }=\overrightarrow{M}\times \overrightarrow{B}$, equivalent to a force applied on the tip normal to the beam surface^65^. The drawbacks of this method are that magnetic coated cantilevers have to be prepared on purpose and that the magnetic coating is easily damaged, especially in liquids. In Fig. 9(b), a modulated current is driven in a triangular cantilever in the presence of a static magnetic field. In this case a Lorentz force on the tip is generated by the modulated current^66^ A different approach is based on the photothermal effect. The photothermal excitation is applied to the base of the cantilever via a modulated laser beam that is partially absorbed by the metallic coating as shown in Fig. 9(c). The thermal stresses caused by light absorption apply a bending moment to the beam, which is the same as applying an external force on the tip^67^. All the methods described so far apply forces on the tip or on the cantilever’s beam without moving the cantilever base, which is important to avoid acoustic excitation in fluids. Modulating the excitation forces at the resonance frequency results in a stable and nearly ideal cantilever excitation in liquids, as used in standard tapping mode techniques.

To measure the viscoelastic constant for each pixel in a raster scanning measurement, the excitation impulse must be applied to the cantilever starting from a known equilibrium position (see Fig. 2b). In practice may be simpler to adopt a two-pass imaging technique analogous to the widely used ‘lift mode’ (which is employed in magnetic and electric force microscopy). The first pass is the normal AM imaging pass, acquiring the topography of the sample. The second pass is at a fixed relative height above the surface. During the second pass the cantilever is excited by an impulsive sinc force at every pixel position and the response recorded. In this way, the dynamical properties of the interacting cantilever and the Q values can be obtained on a pixel-by-pixel basis.

The impulsive excitation on the cantilever can be implemented using one of the described methods by modulating the applied force using a digitally synthesized signal in the form of a sinc temporal profile, as in Fig. 2(a). A digital oscilloscope or a time digitizer electronics can be used to record the deflection signal, eliminating altogether the need for feedback loops, amplitude or frequency stabilization and the electronics that usually complement a standard high level AFM instrument.

The bandwidth for the excitation system depends on the maximum excitation frequency. For a fundamental cantilever’s frequency of 20 kHz and avoiding the direct excitation of the second flexural mode, a sinc with a bandwidth cut off of 50 kHz appears reasonable. Thus the excitation system should cover a maximum bandwidth of 50 kHz, which is fully into the possibilities of modern AFM instruments. Once the cantilever is excited, the nonlinear impact dynamics usually excites higher flexural modes. To estimate the minimum bandwidth for detection we take into account the possibility of exciting up to the third flexural mode (17.55*f*_1_ = 351 kHz). The minimum detection bandwidth needed to satisfy the Nyquist sampling theorem for all the excited modes is 700 kHz. Such a sampling frequency is amply exceeded in modern instruments. Thus the requirements to apply the impulsive methods of excitation and detection are fully compliant with commercial AFM available today.

## Electronic supplementary material

Supplement material
