# Supplementary material for: Theory of Single-Impact Atomic Force Spectroscopy in liquids with material contrast
Source: Sci Rep. 2018 May 14;8:7534. doi: 10.1038/s41598-018-25828-4 (PMC5951954; doi:10.1038/s41598-018-25828-4)
Supplement: Supplementary file 1 — Supplement material [file 41598_2018_25828_MOESM1_ESM.pdf]

Supplementary Information

Theory of Single-Impact Atomic Force Spectroscopy in liquids with material contrast

Enrique A. López-Guerra[1], Francesco Banfi[2,3], Santiago D. Solares[1], and Gabriele Ferrini[2,3]

[1]Department of Mechanical and Aerospace Engineering, The George Washington University, Washington DC 20052, USA

[2]Interdisciplinariy Laboratories for Advanced Material Physics, Università Cattolica del Sacro Cuore, I-25121 Brescia, Italy

[3]Dipartimento di Matematica e Fisica, Università Cattolica del Sacro Cuore, I-25121 Brescia, Italy

Details of viscoelastic contrast

In the following lines, we offer a more in-depth discussion of the viscoelastic contrast seen through the observables provided by the wavelet analysis technique: amplitude, phase and frequency shifts ($\Delta A, \Delta\phi, \Delta f,$ respectively). These observables result from the single tip-sample impact when the AFM probe is subjected to an impulsive excitation (see more details in the Results section of the main manuscript). For the Standard Linear Solid (SLS), the relation between Laplace transformed shear stress ($\bar{\sigma}(s)$) and amount of transformed shear ($\bar{\varepsilon}(s)$) is:

$$\bar{\varepsilon}(s) = \left[\frac{J_e + J_g \tau s}{1 + \tau s}\right] \bar{\sigma}(s) \tag{S1}$$

where $J_g = 1/G_g$ is the glassy compliance (inverse of the glassy modulus: G_g), which describes the response of the viscoelastic model at infinitely short loading times (or infinitely high loading frequencies); $J_e = 1/G_e$ is the equilibrium or rubbery compliance (inverse of the rubbery modulus: G_e), which describes the response at infinitely long times (or infinitely low loading frequencies); τ refers to the single retardation time of the SLS model. The expression in brackets in Eq. S1 is known as the retardance, and can be thought as the transfer function connecting the transformed stress and strain. In a more general case, the relation between stress and strain in the complex plane may be related through rheological models connecting an arbitrary number of characteristic times (see Figure S1). The model in Figure S1 is

known as the Generalized Voigt Model [1], and includes the SLS which is the specific case where only one retardation time is present (only three parameters are included).

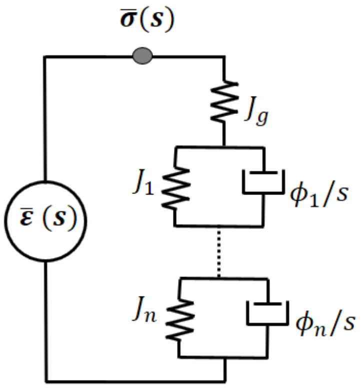

Figure S1 Generalized Voigt Model connecting the Laplace transformed shear stress ($\bar{\sigma}(s)$) and amount of transformed shear ($\bar{\varepsilon}(s)$). This model contains an arbitrary 'n' number of retardation times, being able to represent the physics of the deformation of real history-dependent samples. The SLS model is included in this generalized sample, corresponding to the limiting case where only one retardation time is present, therefore only three parameters are included (J_g, J_1, ϕ_1). Note that the ratio J_n/ϕ_n corresponds to the nth retardation time: τ_n. The symbols J and ϕ represent spring compliance and dashpot fluidity, respectively; they are the inverse of spring modulus G and dashpot fluidity η.

The following discussion is framed on the context of linear viscoelastic behavior. Figure S2 shows dissipated energy (E_{diss}) as function of the material's characteristic time (τ) and the glassy modulus (G_g). This quantity (E_{diss}) is important in viscoelastic characterization because a distinct property of viscoelastic materials is that upon deformation they store but also dissipate energy. Observing viscoelastic behavior requires, however, the condition that the characteristic time of the material (τ) be close to the characteristic timescale of the experiment τ_{exp}. In harmonic excitation experiments (e.g., dynamic mechanical analysis), where force and displacement develop a single sinusoidal behavior, the SLS model shows maximum dissipation when the harmonic frequency of the excitation (ω) is equal to 1/ τ. This can be demonstrated as follows. The dissipated energy is proportional to the loss compliance ($J''(\omega)$) according to [1]: $E_{diss}(\omega) = {\sigma_0}^2\pi J''(\omega)$, where σ_0 is the amplitude of the stress sinusoidal excitation given to the viscoelastic material. The loss compliance for the SLS model in Figure 1 (main manuscript) is $J''(\omega) = \frac{\omega\tau/G}{1+\omega^2\tau^2}$. Then, obtaining the frequency that maximizes energy dissipation in steady-state harmonic excitations implies equating to zero the derivative of dissipated energy with respect to ω and solving for ω (i.e., $\frac{d[E_{diss}(\omega)]}{d\omega} = 0$). By performing the above one finds that the frequency that maximizes

energy dissipation (in steady-state single harmonic excitation experiments) is $\omega = 1/\tau$. Regarding the response's frequency as the inverse of the experimental time $\omega = 1/\tau_{exp}$ implies that, in this case, $\tau_{exp} = \tau$, demonstrating our first assertion that observing viscoelastic behavior implies proximity of experimental and material's characteristic timescales (i.e., when $\tau_{exp} = \tau$ maximum dissipation occurs)

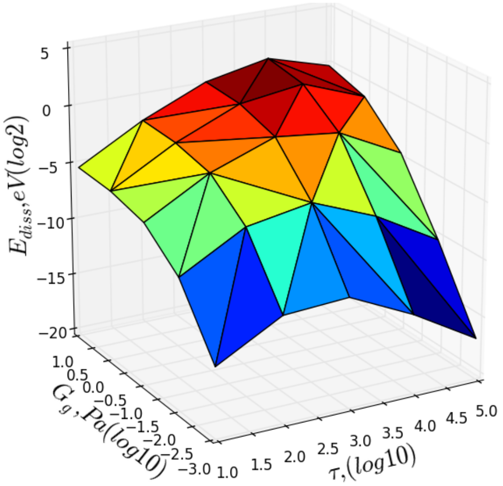

Figure S2 Dissipated energy as function of glassy modulus (G_g) and sample's characteristic time (τ). $\tau_1 = 0.01/\omega, \tau_2 = 0.1/\omega, \tau_3 = 1/\omega, \tau_4 = 10/\omega, \tau_5 = 100/\omega$.

Although our exercise is far from being described by single harmonic response, the above scenario still provides a qualitative explanation of why dissipation is lower when the characteristic time of the material is far from the experimental timescale. For qualitative purposes we may regard our experimental time in our ultra-short experiment as $\tau_{exp} \sim 1/\omega$, being ω the cantilever's first mode resonance frequency. We may regard it roughly as the inverse of the experimental timescale for qualitative purposes because it is the one that governs the cantilever dynamics. However, because this simulation is performed in a low Q environment, the higher spectral content of the tip trajectory cannot be neglected.

Following with our qualitative description we can see in Figure S2 that traveling along iso-G_g lines there is more dissipation towards the center, which corresponds to the point where the material's characteristic time approaches the timescale of the experiment. On the other two extremes the dissipation is smaller, which means that the material displays a more elastic-like behavior and is expected to drop to zero dissipation as it gets farther away (this is beyond the range of material's characteristic times studied). Traveling along iso- τ lines one observes that dissipation grows monotonically, which is attributed to the

fact that at larger glassy modulus of the material, it is expected to have larger tip-sample interactions which favor larger dissipation since the latter is calculated as: $E_{diss} = \int_0^T F_{ts}\dot{z}dt = T < F_{ts}\,\dot{z} >$.

Figure S3a shows the phase shift as function of τ and G_g. Comparing Figure S2 and Figure S3a it can be inferred that the phase shift in the material grid does not follow the trends in dissipated energy. Instead, it seems to follow qualitatively the trend of the average tip-sample forces ($< F_{ts} >$), see Figure S3b. This is counterintuitive based on the standard theory of tapping mode AFM in high Q environments, where it is established that the frequency shift is proportional to energy dissipation [2, 3]. However, for low Q environments Melcher et. al [4] found that the primary source of phase contrast came from the elastic tip-sample interactions which are the ones that govern the momentary excitation of higher modes. They attribute as primary sources of phase contrast: i) tip-sample dissipation and ii) energy transferred from 1st to higher cantilever eigenmodes. In liquids, however, they found —through numerical simulations— that the main contributor is the energy flow from the 1st eigenmode to higher eigenmodes, which is influenced primarily by the tip-sample interactions. According to the above evidence, it is not too surprising to see the match between the maps of frequency shift and $< F_{ts} >$.

Fig S2b shows that —regardless τ— there is a monotonic increase in $< F_{ts} >$ as a function of G_g, which is expected, since larger glassy moduli implies larger tip-sample forces. On the other hand, when analyzing the behavior of the observables as function of τ (following iso-G_g modulus lines) the discussion is more involved. On the extremes of very low or large τ the material behaves in an elastic manner, since as previously discussed, experimental timescale (τ_{exp}) is far from τ, which is contrary to the condition required to observe viscoelastic behavior. In these extremes, it is expected that no energy dissipation occurs and that the material behaves in an elastic-like fashion.

In the extreme where τ_{exp} is large with respect to the material's characteristic time (or τ is very short, as in our case) we can apply final value theorem [1] and infer that the material behaves in a soft-elastic way, with a constant stiffness known as equilibrium or rubbery modulus (G_e):

$$\lim_{\omega \to 0} G^*(\omega) = G_e$$

being $G^*(\omega)$ the complex modulus (the material function describing the response of the material to a harmonic excitation in the steady-state). This qualitatively explains why we see lower average tip-sample forces for low τ values, although in the range of parameters studied we didn't observe a completely elastic-like behavior, which would have resulted in zero dissipation.

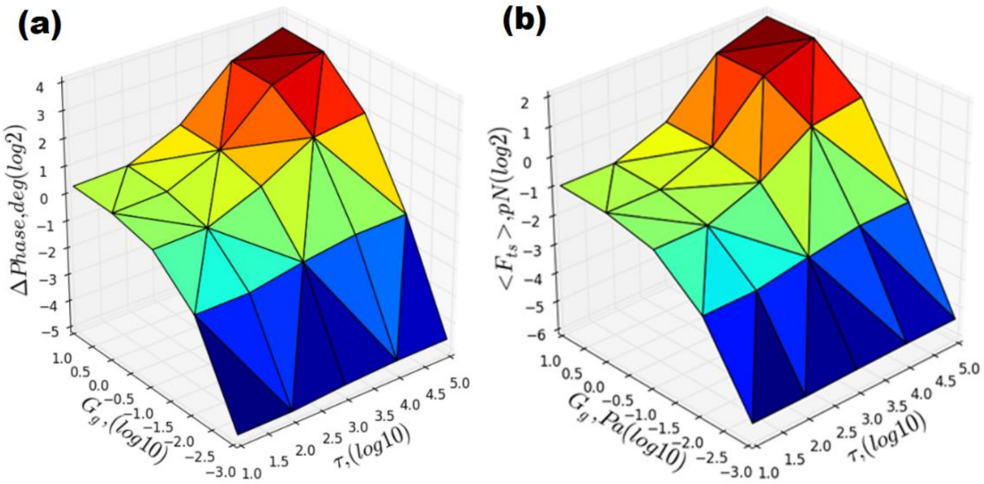

Figure S3 **a)** Phase shift and **b)** average of tip-sample force as functions of glassy modulus (G_g) and sample's characteristic time (τ).

On the other extreme, where τ is large with respect to τ_{exp} (alternatively, τ_{exp} being infinitesimally short), the material also behaves in an elastic-like manner, but being described in its deformation by the glassy modulus G_g. Here, finally the meaning of glassy modulus is given and, as physically intuitive explanation, it is useful to picture the SLS model in Figure S1 being deformed at very high loading rates, which would intuitively result in the inactivity of the dashpot (therefore leading to no dissipation). Formally, the above is proven by applying the initial value theorem [1] which yields:

$$\lim_{\omega \to \infty} G^*(\omega) = G_g$$

The above qualitatively explains what we observe by traveling on an iso-G_g trajectory along the direction of growing τ values (Figure S3b). As expected, the material behaves in a stiffer way (G_g is orders of magnitude higher than G_e in real experiments), which results in higher average tip-sample forces. Of course, there is also a competing effect because these larger forces are accompanied by lower penetrations and lower contact times which have the effect of lowering the average of tip-sample interaction forces. However, the effect of the magnitude of the tip-sample forces seems to dominate over the contact times, resulting in an overall increase of $< F_{ts} >$ as G_g increases.

Clearly, these extremes (infinitely low and large experimental times) are not reached in the range of Figure S3b, since in these ranges the material is still viscoelastic which is evidenced by the values of dissipation given in Figure S2. Note that, even though the values of dissipation are smaller in the extremes of the τ axis, they are not negligible.

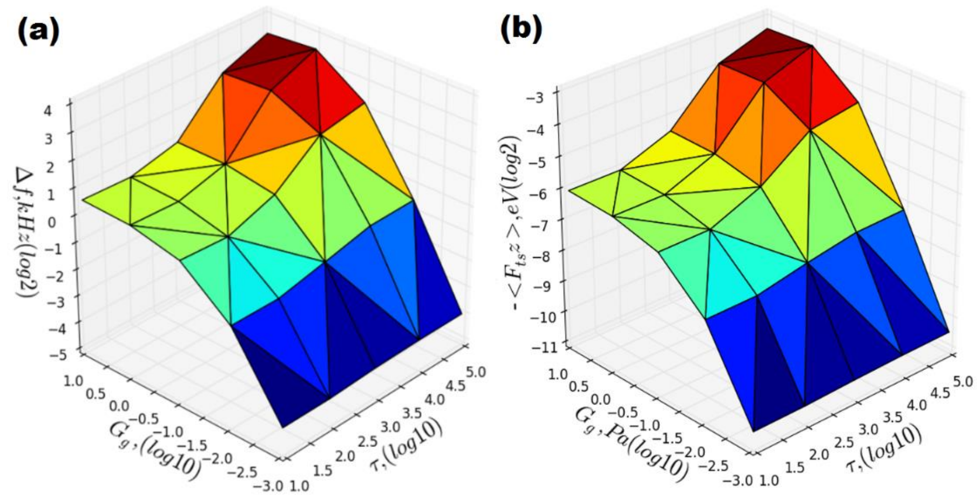

Figure S4 **a)** Frequency shift and **b)** negative of virial as functions of glassy modulus (G_g) and sample's characteristic time (τ)

The frequency shift is often associated with the average of the tip-sample interaction force gradient (at least in the context of tapping mode in air [5]). Here, we observe that it follows the behavior of the negative of the virial (see Figure S4). These trends seem close to the ones discussed in Figure S3, and our qualitative explanations previously provided apply also to the current context. However, the reason why we associate the frequency shift with the virial (Figure S4) and the phase shift with the average tip-sample force (Figure S3) is more apparent when analyzing Figure 5 carefully (see main manuscript) and Figure S5. Following iso-τ lines in Figure 5c demonstrates that the phase shift grows monotonically (for the range of materials studied here) as G_g grows (this is also evidenced in Figure S3a). It can also be seen that following iso-G_g lines in Figure 5f, phase shift also grows monotonically as τ increases (this can also be seen in Figure S3a). The above is consistent with the behavior of $< F_{ts} >$ and can be clearly seen in Figure S5b.

Now turning to Figure 5b, we can follow iso-G_g lines (Figure 5d in the main manuscript) and observe that the frequency shift increases monotonically as τ increases. However, by analyzing iso-τ lines (in Figure 5b in the main manuscript) we note that the frequency shift also increases but not in monotonic manner for $\tau = 10/\omega$ and $\tau = 100/\omega$. This behavior is consistent with the behavior of the (negative) viral. The above is more clearly seen in Figure S5a. Although the information given by Figure S5a and Figure S5b seems to be qualitatively equivalent, a closer look allows to identify distinctly a resemblance between the phase shift and $< F_{ts} >$, and a resemblance between the frequency shift and the viral.

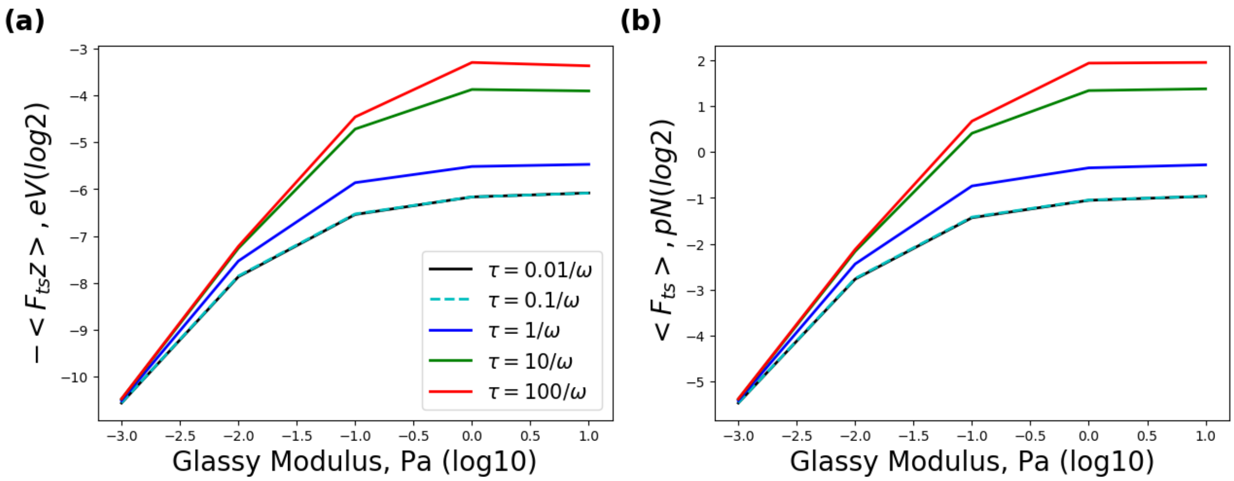

Figure S5 **a)** Virial as function of G_g from different τ values. For $\tau = 10/\omega$ and $\tau = 100/\omega$, virial does not grow monotonically with G_g (the maximum for both is achieved for G_g = 1.0 GPa). This behavior is qualitatively consistent with the behavior of the frequency shift (see Figure S4b). **b)** Average tip-sample force as function of G_g, $< F_{ts} >$ grows monotonically with G_g, a behavior qualitatively consistent with the behavior of phase shift (Figure S2).

REFERENCES

1. Tschoegl, N.W., *The phenomenological theory of linear viscoelastic behavior: an introduction*. 2012: Springer Science & Business Media.
2. Cleveland, J., et al., *Energy dissipation in tapping-mode atomic force microscopy.* Applied Physics Letters, 1998. **72**(20): p. 2613-2615.
3. Tamayo, J. and R. García, *Relationship between phase shift and energy dissipation in tapping-mode scanning force microscopy.* Applied Physics Letters, 1998. **73**(20): p. 2926.
4. Melcher, J., et al., *Origins of phase contrast in the atomic force microscope in liquids.* Proceedings of the National Academy of Sciences, 2009. **106**(33): p. 13655-13660.
5. Garcıa, R. and R. Perez, *Dynamic atomic force microscopy methods.* Surface science reports, 2002. **47**(6): p. 197-301.
